# Combination of Vaccine With IL‐12‐Armed Oncolytic Virus SKV‐012 Synergistically Potentiates Immune Responses in HPV‐Associated Malignancies

**DOI:** 10.1002/mco2.70737

**Published:** 2026-04-13

**Authors:** Nian Yang, Long Xu, Meijun Zheng, Huaqing Lu, Yongdong Chen, Zhixiong Zhu, Wanqin Zeng, Zeng Wang, Hexian Li, Jia Li, Zheng Jiang, Pingfu Zeng, Guoqing Wang, Hai Xie, Zongliang Zhang, Hui Yang, Aiping Tong

**Affiliations:** ^1^ State Key Laboratory of Biotherapy and Cancer Center Research Unit of Gene and Immunotherapy Chinese Academy of Medical Sciences Collaborative Innovation Center of Biotherapy West China Hospital Sichuan University Chengdu Sichuan China; ^2^ Department of Otolaryngology‐ Head & Neck Surgery West China Hospital Sichuan University Chengdu Sichuan China; ^3^ Department of Biotherapy Cancer Center West China Hospital Sichuan University Chengdu Sichuan China; ^4^ Department of Ophthalmology West China Hospital Sichuan University West China Medical School Chengdu Sichuan China; ^5^ Frontiers Medical Center Tianfu Jincheng Laboratory Chengdu Sichuan China

**Keywords:** antitumor responses, HPV‐associated malignancies, oncolytic viruses, therapeutic vaccines, tumor microenvironment

## Abstract

Currently, patients with advanced‐stage or refractory human papillomavirus (HPV)‐associated malignancies have few therapeutic options. Despite therapeutic HPV vaccines having been investigated, the lack of appreciable efficacy highlights the urgent need to develop more effective strategies. Here, we developed an immuno‐oncotherapy for HPV‐induced tumors based on an adenoviral (Ad)‐vectored therapeutic vaccine that contains concatemeric T cell epitopes, and evaluated oncolytic viruses (OV) as potential approach to enhance vaccine efficacy. We observed that the therapeutic vaccine encoding the HPV E7 oncoprotein epitope (Ad‐E7P) significantly inhibited tumor growth in HPV‐induced murine models by inducing systemic antitumor CD8+T cell responses and promoting the formation of tertiary lymphoid structures in peritumoral regions. We then evaluated the potential of combining the vaccine with an interleukin‐12 (IL‐12)–armed oncolytic herpesvirus (SKV‐012) in preclinical models. The combination therapy elicited potent antitumor responses by inducing antigen‐specific T‐cell expansion, remodeling the tumor microenvironment, and generating immune memory, which led to tumor clearance. Overall, these findings support that the vaccine synergizes with the OV as an effective approach to enhance antitumor immunity in HPV‐associated malignancies.

## Graphical abstract

A synergistic immunotherapeutic platform based on the intratumoral delivery of oncolytic virus and intramuscular vaccination activates both local and systemic antitumor immunity. This synergistic approach expands the universality and therapeutic efficacy of vaccines in HPV‐associated malignancies. It enables long‐term clearance by generating adaptive immune memory, highlighting myriad opportunities for cancer therapy.

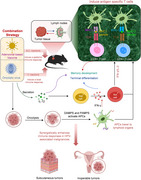

## 1 Introduction

Human papillomavirus (HPV) infection with high‐risk subtypes drives tumorigenesis in the majority of cervical, oropharyngeal, anal, and vulvar cancers, among others [1, 2]. While surgery, radiation, and chemotherapy achieve high cure rates for early‐stage human papillomavirus–positive oropharyngeal squamous cell carcinoma (HPV‐OPSCC), there remains an unmet need for new therapeutic alternatives for patients with unresectable and/or recurrent HPV‐related tumors [3]. During the past decade, vaccines have revolutionized the treatment of cancers. Therapeutic vaccines designed to elicit tumor‐specific T‐cell responses have shown considerable clinical benefits in HPV‐related cancers [4, 5]. VGX‐3100, which encodes optimized synthetic consensus E6 and E7 antigens, is currently the only therapeutic HPV vaccine being investigated in a Phase III clinical trial [6, 7].

Preclinical and clinical studies have demonstrated promising outcomes for Ad‐based vaccines, such as those against severe acute respiratory syndrome coronavirus 2 (SARS‐CoV‐2) and the Ebola virus [8, 9]. Furthermore, Ad‐based vaccine platform was developed for cancer therapy due to its ability to effectively deliver DNA encoding antigens or epitopes, promote endogenous antigen production, and activate both humoral and cellular immune responses [10]. Targeting both E6 and E7 antigens is a promising therapeutic approach, as these proteins are consistently expressed in HPV‐associated cancers [6, 11, 12]. However, cancer vaccines as monotherapies have displayed limited success. It is also important to recognize that relying solely on vaccine therapy cannot fully overcome all the challenges encountered in immunotherapy. Multiple phase III trials in advanced tumors have reported negative outcomes [13, 14, 15]. Additionally, tumor immune evasion and resistance mechanisms further hamper vaccine efficacy. Numerous strategies are being investigated in preclinical studies to improve the potency of cancer vaccines [16, 17]. Previous studies have demonstrated that combining an oncolytic virus (rVSV‐LCMV‐G) with an mRNA vaccine significantly reduces tumor burden and prolongs survival, exhibiting synergistic efficacy [].

In contrast to the systemic immune activation elicited by vaccines, OV provoke a localized in situ vaccination effect [19], inducing tumor‐specific adaptive immune responses and potentially serving as promising adjuncts to amplify the anti‐tumor efficacy of cancer vaccines [20, 21]. Additionally, OV can be genetically engineered to enhance antitumor immune responses [19, 22]. Interleukin‐12 (IL‐12) is a widely used immunomodulator for improving the efficacy of OV or vaccines, as it effectively induces adaptive Th1‐type responses, enhances antigen processing, and reprograms immune‐excluded or immune‐desert tumor microenvironments [23, 24, 25]. However, systemic administration of IL‐12 is associated with severe toxicity. In this context, localized delivery of IL‐12 via OV may serve as an effective adjuvant for vaccines, potentially overcoming the limitations of monotherapy.

Here, we report the results of the adenoviral‐vectored peptide vaccine as monotherapy and in combination with IL‐12‐loaded oncolytic virus SKV‐012 in preclinical models. We found that this combination synergistically enhances antigen‐specific T‐cell responses, inducing durable and broad‐based immune responses that inhibit tumor growth and prolong survival in HPV‐related mouse models. These findings suggest a novel therapeutic approach for HPV‐associated cancers.

## 2 Results

### 2.1 Antitumor Efficacy of an adenovirus‐based Therapeutic HPV Vaccine

**FIGURE 1:**
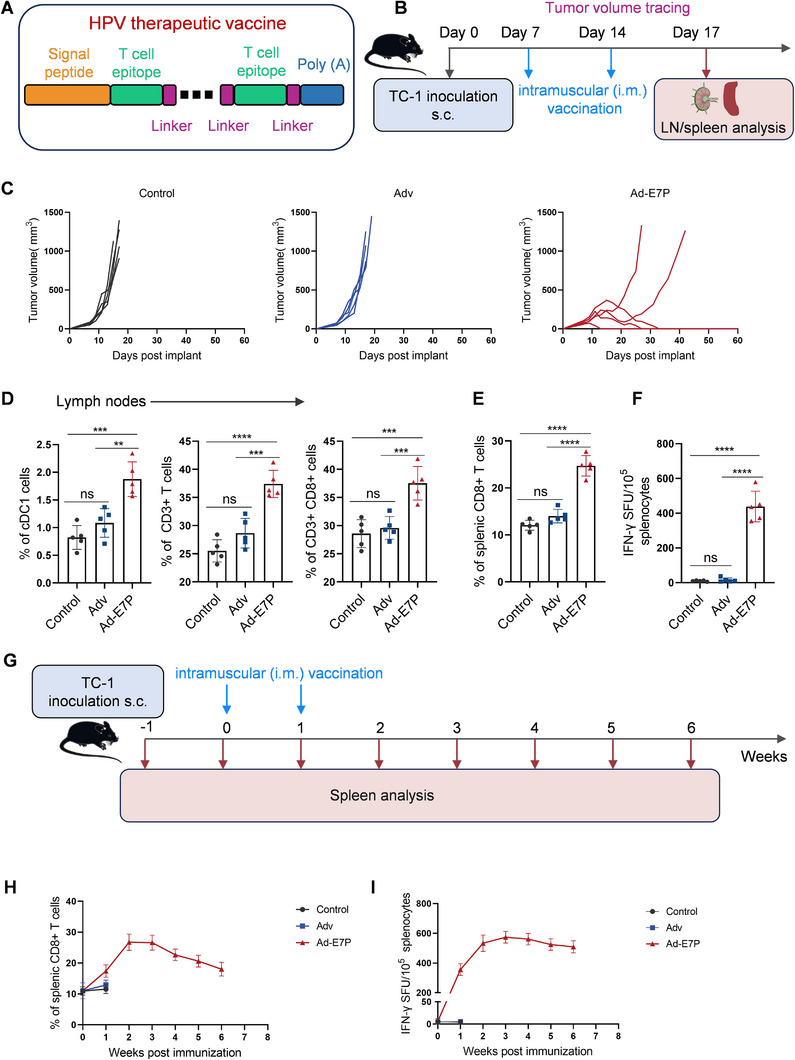
Ad‐E7P therapeutic vaccine induces antigen‐specific immune responses and potent antitumor protection in TC‐1 tumor model. (A) Schematic of the adenoviral‐based therapeutic vaccine. (B) Experimental schema for evaluating the antitumor efficacy of Ad‐E7P in the TC‐1 tumor model. C57BL/6 mice were subcutaneously inoculated with 1 × 10^6^ TC‐1 tumor cells in the right flank, followed by weekly intramuscular (i.m.) immunizations with 10^9^ viral particles (VP) of Ad‐E7P or the empty vector (Adv) for a total of two doses when tumor volumes reached approximately 50 mm^3^. Mice in the negative control group received PBS. On Day 17, lymph nodes and spleens were harvested for immune cell analysis. (C) Tumor growth curves of mice treated with Ad‐E7P, Adv, or PBS (*n* = 5 mice per group). (D and E) Flow cytometric analysis of DCs, total T cells, and CD8+ T cells in the lymph nodes (D), and CD8+ T cells in the spleen (E) following immunization (*n* = 5 mice per group). (F) On Day 17, antigen‐specific T cell activation in splenocytes was assessed by ELISpot assay following in vitro stimulation with the E7_49‐57_ peptide. SFU, spot‐forming unit (*n* = 5 mice per group). (G) Treatment schedule for Ad‐E7P vaccination and correlative immune kinetics analysis. (H and I) Flow cytometric analysis of the percentages of CD8+ T cells (H) and ELISpot analysis of IFN‐γ–producing cells (I) in the spleen at different time points following Ad‐E7P vaccination (*n* = 3 per group). Data are presented as the means ± SD. One‐way analysis of variance (ANOVA) with Tukey's multiple comparisons test was performed for all comparisons (*p < 0.05, **p < 0.01, ***p < 0.001, ****p < 0.0001). *ns*, not significant.

To assess the therapeutic potential of an adenovirus‐based vaccine in HPV‐related tumors, we designed an Ad‐vectored peptide vaccine, Ad‐E7P, based on a previously reported murine H2‐Db‐restricted CD8+ T cell E7 epitope (amino acids [aa] 49 to 57). E6 was excluded due to its lack of immunogenicity in the murine model as mentioned previously [26]. The epitope was arranged in a linear tandem format, and separated by short peptide spacers to optimize proteasomal cleavage and subsequent presentation on MHC class molecules (Figure 1A). Next, we evaluated the antitumor effects and immune responses of intramuscular (i.m.) injections of the Ad‐E7P vaccine in mice bearing subcutaneous TC‐1 tumors that express the HPV16 E6 and E7 antigens. The results showed that the Ad‐E7P‐treated group exhibited stronger tumor growth inhibition than the adenovirus empty vector (Adv) group or the control group, suggesting a more effective antitumor response (Figure 1B,C). We hypothesized that the antitumor effects were driven by the antigen‐specific epitopes included in the vaccine. Therefore, we evaluated the changes in and activation of dendritic cells (DCs) and various T cell subsets after 10 days vaccination. We observed that immunization with Ad‐E7P moderately increased the proportion of cDC1, which are known to cross‐present antigens to MHC class I molecules and promote the effective activation of CD8+ T cells in lymph nodes and spleen (Figure 1D). The percentages of CD3+ and CD8+ T cells in lymph nodes and spleen significantly increased after two doses of vaccination in the Ad‐E7P group (Figure 1D,E). Similarly, we also observed higher numbers of E7‐specific CD8+ T cells in Ad‐E7P‐vaccinated mice compared with Adv‐ or PBS‐treated mice by using E7‐specific tetramer staining (Figure S7A,S7B). Additionally, splenocytes from the Ad‐E7P‐treated group exhibited increased interferon‐γ (IFN‐γ) production, as determined by enzyme‐linked immunospot (ELISpot) assay, indicating effective activation of antigen‐specific T cells (Figure 1F). Results showed that the magnitude of the CD8+ T cell response is highly dependent on the T cell epitope included in the Ad‐E7P vaccine (Figure 1D,F).

Next, we monitored the time course of accumulation of immune response during the vaccine approach (Figure 1G). In the Ad‐E7P group, CD8+ T cells in the spleen increased to peak levels at 2 weeks post‐immunization and then slightly decreased between 4 and 6 weeks after vaccination. IFN‐γ production against the E7 antigen gradually increased at 2–3 weeks post‐immunization (Figure 1H,I). The control and Adv groups were monitored for 1‐week post‐immunization due to tumor growth approaching ethical limits. Taken together, these findings preliminarily demonstrate that the Ad‐E7P vaccine exhibits tumor‐controlling effects and effectively induces antigen presentation and T cell activation.

### 2.2 Ad‐E7P vaccine Induces Antigen‐specific Immune Responses Within the Tumor Microenvironment (TME) and Confers Protective Immunity

**FIGURE 2:**
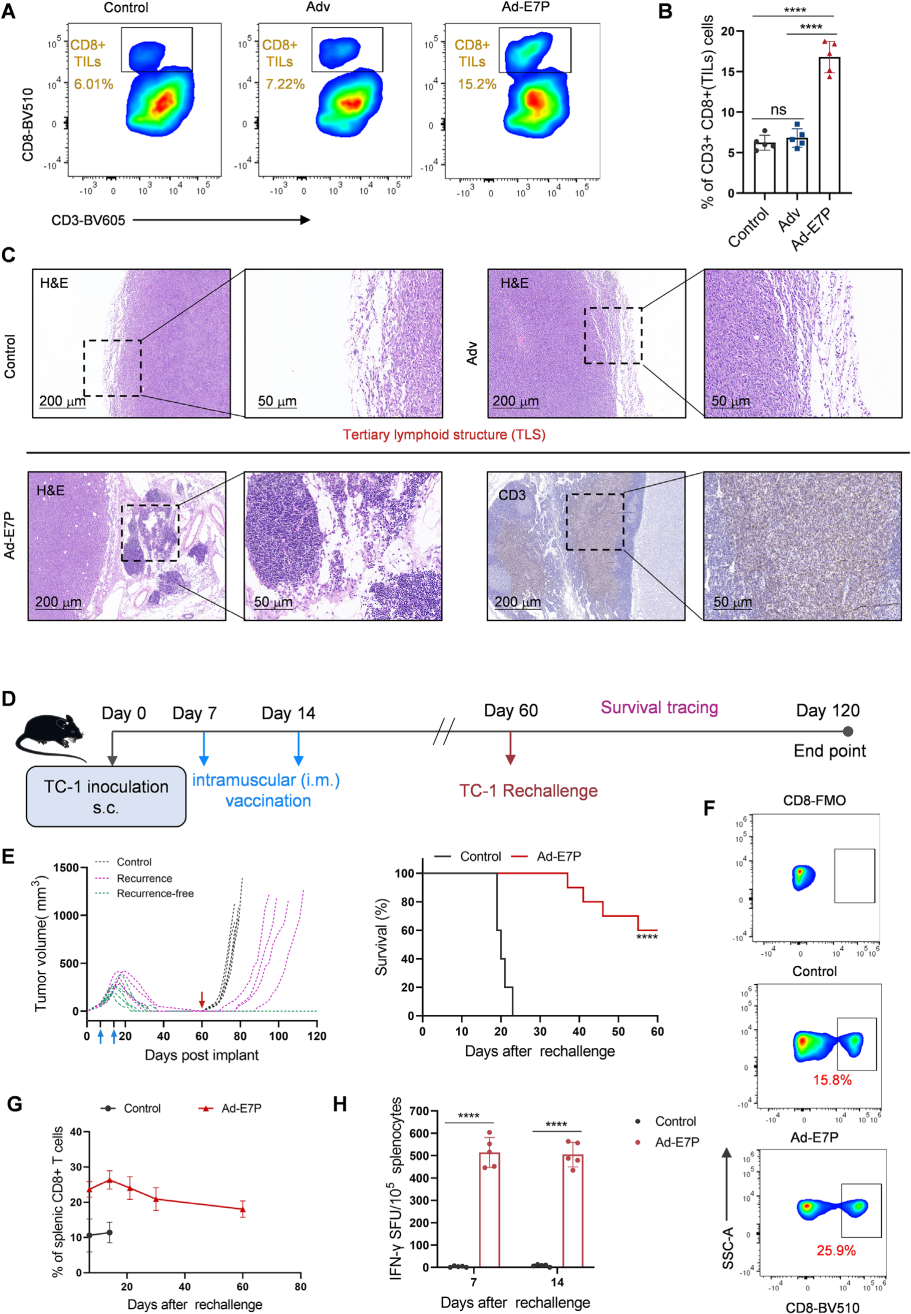
The therapeutic vaccine Ad‐E7P induces intratumoral immune activation and reduces tumor recurrence. (A) Representative flow cytometry plots of CD8+ TILs. Numbers indicate the percentage of CD8+ TILs within the gated population. (B) Quantification of CD8+ TILs is shown (*n* = 5 per group). (C) Representative hematoxylin and eosin (H&E)–stained images of paraffin‐embedded TC‐1 tumor sections from different treatment groups. TLSs were observed adjacent to tumors in Ad‐E7P‐vaccinated mice and confirmed by both H&E and CD3 immunohistochemical staining. Scale bars: 200 µm (overview) and 50 µm (zoomed‐in view). (D and E) Experimental scheme. TC‐1 tumor‐regressing mice, cured by Ad‐E7P vaccination, were re‐inoculated with the same number of tumor cells in the left flank on day 60, and survival was monitored (D). Untreated mice served as controls. Tumor growth curves (E, left) and corresponding survival kinetics of mice after tumor rechallenge (E, right) (Control, *n*  =  5 mice; Ad‐E7P, *n*  =  10 mice). (F and G) Flow cytometric analysis of splenic CD8+ T cells after tumor rechallenge. Representative plots from Day 7, including a CD8 fluorescence‐minus‐one (FMO) control to identify the positive population (F), and the percentages of CD8+ T cells on Days 7, 14, 21, 30, and 60 (G) (*n* = 3 per group). (H) Summary statistics for ELISpot assays on Day 7 and 14 after tumor rechallenge (*n* = 5 per group). Data are presented as mean ± SD. r. Statistical analyses were conducted by one‐way ANOVA with Tukey's correction for multiple comparisons in (B), by two‐way ANOVA in (H), and by log‐rank (Mantel‐Cox) test in (E). *p < 0.05, **p < 0.01, ***p < 0.001, ****p < 0.0001). *ns*, not significant.

To further validate these findings, we next investigated the ability of Ad‐E7P–elicited CD8+ T cells to infiltrate the tumor bed. Two intramuscular injections of Ad‐E7P resulted in increased infiltration of total CD8+ tumor‐infiltrating lymphocytes (TILs) (Figure 2A,B) and E7‐specific CD8+ T cells (Figure S7C,S7D). However, no significant differences were observed between the Adv group and the control group (Figure 2A,B). Notably, hematoxylin and eosin (H&E) staining revealed the presence of tertiary lymphoid structures (TLSs) adjacent to the tumors in the Ad‐E7P–treated group. Immunohistochemical (IHC) staining further confirmed the presence of a large number of T cells within the TLSs induced by Ad‐E7P (Figures 2C and S8). In contrast, no TLSs were observed in the control or Adv groups, suggesting that the immune responses were likely specifically induced by the vaccine (Figure 2C).

We further evaluated whether Ad‐E7P could elicit long‐term protective immunity and prevent tumor recurrence. Mice cured by Ad‐E7P vaccination in repeated experiments were used in TC‐1 tumor rechallenge studies, and age‐matched, treatment‐naïve mice were used as controls. Both groups were subcutaneously implanted with identical numbers of TC‐1 tumor cells in the left flank (Figure 2D).

At the time points of primary and rechallenged, Ad‐E7P immunized mice significantly inhibited tumor growth and prolonged survival compared to the control group (untreated), with 60% of mice in the Ad‐E7P group remaining tumor‐free (Figure 2E). Among the mice without tumor recurrence, approximately 50% exhibited early tumor clearance, while the other 50% showed delayed clearance, indicating a complex mechanism of clearance and protection. To understand why tumors relapsed despite vaccination, we analyzed MHC‐I and E7 expression in relapsed tumors collected from the experimental models shown in Figure 2D. Relapsed tumors exhibited reduced MHC‐I (Figure S9A) and E7 (Figure S9B,S9C) expression compared with tumors in the early regression phase, indicating loss of antigen expression and presentation as major mechanisms of immune escape. This observation is consistent with the findings of Sanne Bevers et al., who reported a profound decrease in E7 expression levels after vaccination with E7‐TriMix [26].

To further define the immune response following rechallenge, we analyzed total CD8+ T cells, E7‐specific CD8+ T, and IFN‐γ producing post‐rechallenge. As expected, we observed that, compared to the untreated group, total CD8+ T cells (Figure 2F,G), E7‐specific CD8+ T (Figure S9D), and IFN‐γ producing (Figure 2H) were significantly increased in Ad‐E7P–treated mice. In summary, these findings strongly suggest that the Ad‐E7P vaccine induces robust and long‐lasting T cell‐mediated immunity capable of rapid cytotoxic activity upon antigen re‐exposure.

### 2.3 Combination of Ad‐E7P Vaccine and IL‐12‐loaded Oncolytic Virus Enhances Therapeutic Efficacy Against TC‐1 Tumors

**FIGURE 3:**
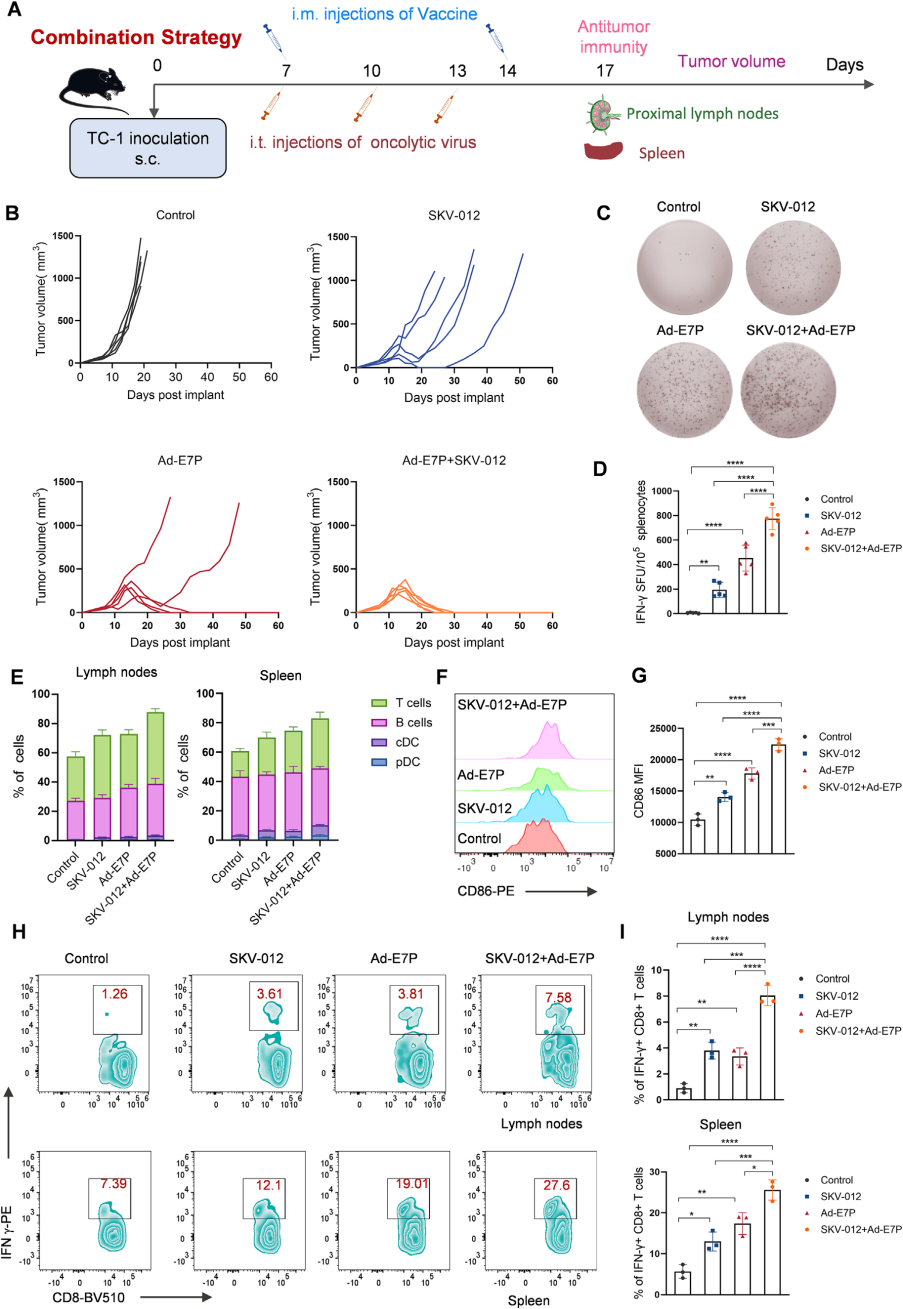
Ad‐E7P vaccine combined with the oncolytic virus SKV‐012 inhibits tumor progression and induces a potent antitumor immune response in the TC‐1 tumor‐bearing mouse model. (A) Experimental scheme. C57BL/6 mice were subcutaneously inoculated with 1 × 10^6^ TC‐1 tumor cells in the right flank. When tumor volumes reached approximately 50 mm^3^, mice were assigned to receive Ad‐E7P, SKV‐012, a combination of Ad‐E7P and SKV‐012, or PBS as a control. For Ad‐E7P group, mice received two doses of 10^9^ VP Ad‐E7P administered once a week. For SKV‐012 group, mice received three intratumoral injections of 10^6^ PFU SKV‐012 every 3 days. For combination treatment group, mice received two doses of 10^9^VP Ad‐E7P once a week and three doses of 10^6^ PFU SKV‐012 intratumorally every three days. (B) Tumor growth curves (*n* = 5 per group). (C and D) Representative IFN‐γ ELISPOT images (C) and summary of ELISPOT results (D) from splenocytes stimulated in vitro with the E7_49‐57_ peptide (*n* = 5 per group). (E) Proportions of various immune cell populations in lymph nodes and spleens (*n* = 3 per group). (F and G) Representative flow cytometric analysis of CD86 expression on dendritic cells in the lymph nodes (F), and quantification of mean fluorescence intensity (MFI) of CD86 expression (G) (*n* = 3 per group). (H and I) Representative flow cytometric analysis of IFN‐γ+ CD8+ T cells in both lymph nodes and spleen (H), and statistical analysis of the results (I). (*n* = 3 per group). Data are presented as mean ± SD. One‐way ANOVA followed by Tukey's multiple comparisons test was used for statistical analysis. (*p < 0.05, **p < 0.01, ***p < 0.001, ****p < 0.0001). *ns*, not significant.

Although Ad‐E7P vaccination demonstrated promising antitumor activity in TC‐1 tumors, it was insufficient to completely prevent tumor relapse (Figure 2E). Broader immune activation is essential to prevent the outgrowth of tumors following antigen escape after vaccination. Previous studies have shown that the IL‐12‐armed oncolytic herpes simplex virus SKV‐012 exerts potent antitumor effects and increases the density of TILs in multiple murine tumor models (e.g., CT26 and B16F10‐HVEM) [27]. We also found that the oncolytic virus SKV‐012 can mediate the killing of HPV‐related tumor cells in vitro, including TC‐1, mEERL, Caski, SiHa, and HeLa (data not shown). Consequently, we explore the combination of the Ad‐E7P vaccine and oncolytic virus SKV‐012 as a potentially synergistic approach to enhance antitumor immunity and overcome the limitations of monotherapy. To test this, we employed the previously described subcutaneous TC‐1 tumor model. Mice received three intratumoral injections of SKV‐012 and two intramuscular injections of 10^9^ VP Ad‐E7P following tumor cell inoculation (Figure 3A). In parallel, three additional groups were treated with either SKV‐012 alone, Ad‐E7P alone, or PBS as a control. As expected, the combination therapy of SKV‐012 and Ad‐E7P significantly reduced tumor burden and achieved tumor‐free survival at the 2‐month follow‐up, compared to either monotherapy (Figure 3B). Moreover, ELISpot assays revealed a higher frequency of antigen‐specific T cells in the combination treatment group compared to the monotherapy and control groups (Figure 3C,D).

To assess the immune‐activating capacity of the combination therapy, we analyzed immune cell populations in both the lymph nodes and spleens using flow cytometry. We observed a significant increase in the frequency of DCs and T cells compared to the SKV‐012 group, Ad‐E7P group, and the control group (Figure 3E). Additionally, we detected upregulated expression of CD86 (Figure 3F,G) and CD40 (Figure S10A) on DCs in the lymph nodes and spleens (Figure S10B), suggesting enhanced antigen‐presenting capacity, which subsequently promoted the priming of tumor antigen‐specific T cell responses. Furthermore, we identified E7‐specific CD8+ T cells in the spleen using E7‐specific tetramers and intracellular staining. MHC‐I tetramer analysis revealed a substantial increase in the number of E7‐specific CD8+ T cells in the spleen at day 17 post‐tumor inoculation (Figure S11A,S11B). Intracellular cytokine staining analysis revealed an approximately two‐fold increase in IFN‐γ+ CD8+ T cells in both the spleen and lymph nodes in the combination group, compared to either SKV‐012 or Ad‐E7P monotherapy (Figure 3H,I).

To further investigate dose‐dependent synergy with the oncolytic virus, we tested two reduced doses (1E+08 and 5E+08) of the Ad‐E7P vaccine with the oncolytic virus antitumor efficacy. We observed that, compared with the vaccine group, combination therapy with reduced vaccine doses significantly inhibited tumor growth in TC‐1 tumor‐bearing mice (Figure S12A,S12B). These results indicate that the combination therapy enhanced the limited efficacy of low‐dose vaccination against HPV‐induced tumors in mice.

### 2.4 Characterization of Immune Cell Infiltration Within TME

**FIGURE 4:**
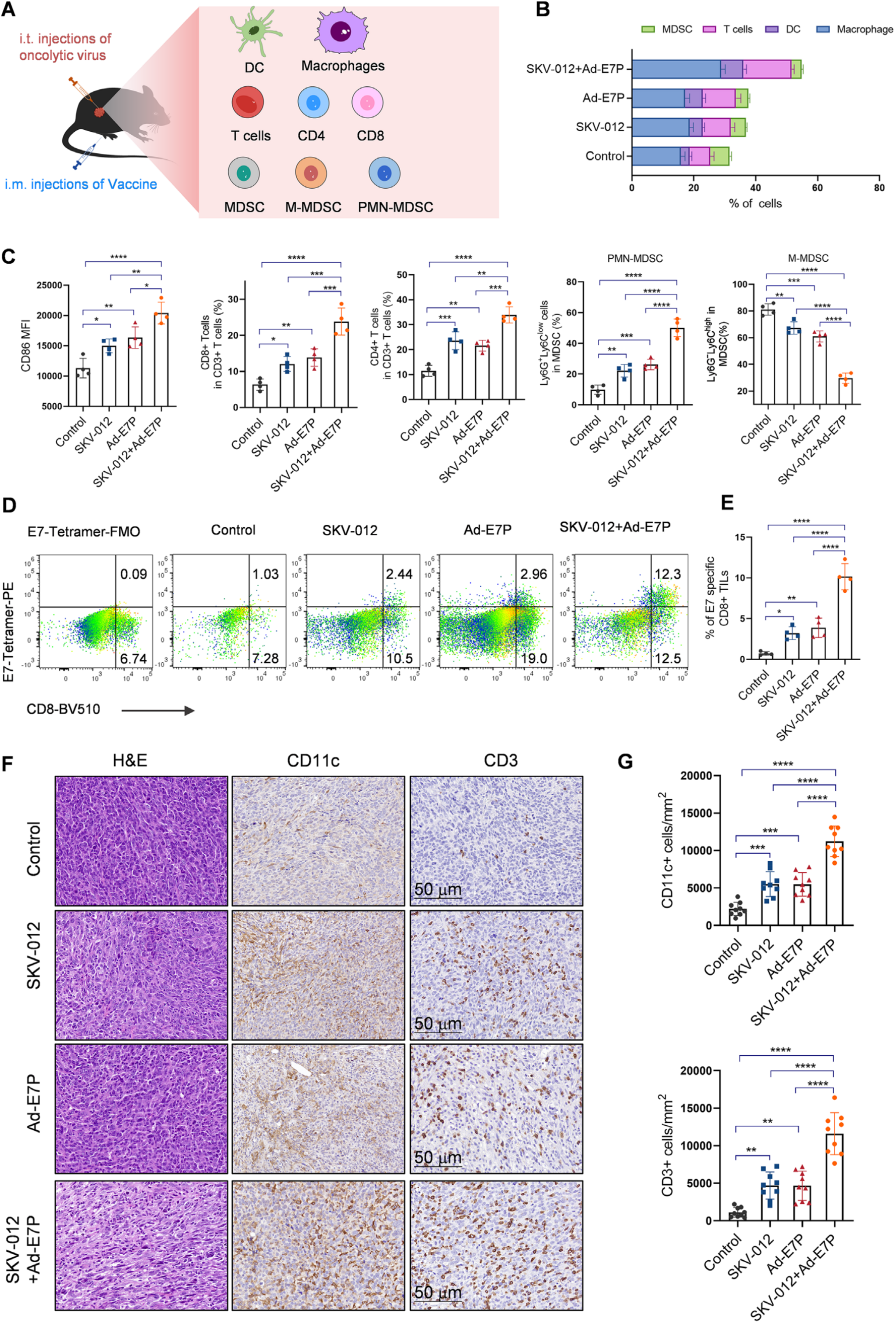
Characterizing immune cell infiltration in the TME. (A) Schematic representation of evaluated immune cell infiltration types within the TME in this research. (B) Proportions of various immune cell populations in tumors on day 17 (*n* = 4 per group). (C) Statistical analysis of CD86 MFI on dendritic cells and proportions of CD8+ T cells, CD4+T Cells, PMN‐MDSCs, and M‐MDSCs in the TME (*n* = 4 per group). (D) Representative flow cytometric analysis of E7‐specific CD8+ T cells within the TME. The E7‐Tetramer FMO control serves to accurately identify the positive population. (E) Quantification of E7‐Specific CD8+ T Cells as Shown in (D) (*n* = 4 per group). (F) H&E staining and IHC staining for CD11c and CD3 in TC‐1 tumor tissue after treatment. Scale bars, 50 µm. (G) Quantification of the CD11c and CD3 cells in the tumors/mm^2^ (*n* = 3 mice per group; *N* = 3 images/field of view per mouse). Data are presented as mean ± SD. One‐way ANOVA followed by Tukey's multiple comparisons test was used for statistical analysis. (*p < 0.05, **p < 0.01, ***p < 0.001, ****p < 0.0001). *ns*, not significant.

**FIGURE 5:**
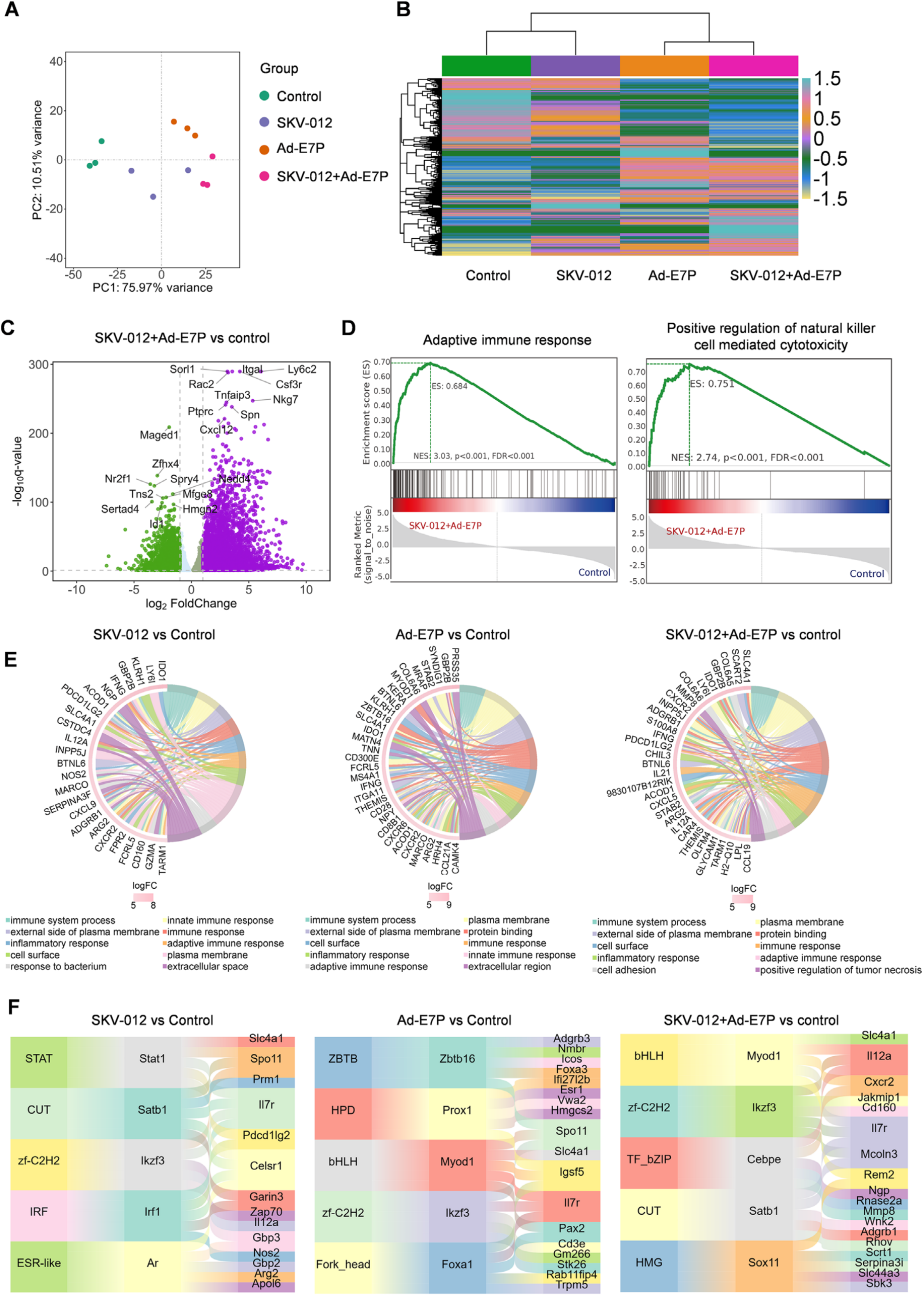
Transcriptomics analysis. (A) PCA plot of RNA‐seq data from TC‐1 tumors harvested on Day 17. (B) Heatmaps of differentially expressed genes among the different groups. (C) Volcano plot displaying differentially expressed genes between the two groups. Green dots represent downregulated genes and purple dots represent upregulated genes. (D) GSEA showing enrichment of pathways between combination group and control group. (E) GO enrichment chord plot showing the top 10 enriched pathways at the bottom. The top 5 genes with the highest |logFC| values in each category are displayed on the left. (F) Sankey diagram illustrating the relationship between differential transcription factors and their target genes. Plot represents the top 5 transcription factors and their corresponding top 5 target genes with the highest |log2FC| values.

To further assess the systemic immune response and its impact on tumor infiltration, we repeated the experiments shown in Figure 3A and harvested TC‐1 tumor tissues on Day 17. Flow cytometric analysis was then performed to evaluate immune cell infiltration within TME (Figure 4A). As expected, the levels of TILs and DCs were higher in the combination treatment group compared to the SKV‐012‐ or Ad‐E7P‐ alone groups and were significantly elevated relative to the control group (Figure 4B). Additionally, the proportion of myeloid‐derived suppressor cells (MDSCs) was reduced in the SKV‐012+Ad‐E7P group. We further evaluated the expression of the activation marker CD86 on DC cells within the tumor tissue. Similar to the findings in the lymph nodes (Figure 3F), an upregulation of CD86 expression was observed (Figure 4C). Next, we further characterized the T cell subsets within the tumor. We observed an increase in both tumor‐infiltrating CD8+ and CD4+ T cells in the combination treatment group, with the proportion of CD8+ T cells nearly one‐fold compared to the monotherapy groups (Figure 5C).

MDSCs exert immunosuppressive effects through various mechanisms, including the upregulation of arginase 1 (Arg1) and inducible nitric oxide synthase (iNOS), which directly inhibit the activities of NK cells, B cells, and T cells. Polymorphonuclear myeloid‐derived suppressor cells (PMN‐MDSCs) and monocytic MDSCs (M‐MDSCs) are distinct MDSC subsets, each employing unique mechanisms to suppress immune responses. M‐MDSCs suppress T cell activation through both antigen‐specific and non‐specific pathways, whereas PMN‐MDSCs predominantly mediate immune suppression in an antigen‐specific manner, promoting tolerance in CD8+ T cells. Despite a reduction in the overall proportion of MDSCs in the treatment group (Figure 4B), further analysis based on Ly6C and Ly6G expression revealed a shift in MDSC subset distribution. Specifically, the treatment group showed an increased proportion of PMN‐MDSCs and a decreased proportion of M‐MDSCs, suggesting that immune suppression in the SKV‐012 + Ad‐E7P group may be primarily driven by antigen‐specific mechanisms (Figure 4B,C). Additionally, E7‐specific CD8+ T cells were identified within the TME using E7‐specific tetramers, revealing a higher proportion of E7‐specific T cells in the combination treatment group compared to other groups (Figure 4D,E).

IHC staining was performed to evaluate the infiltration of CD3+ and CD11c+ cells within TC‐1 tumor tissues from experimental mice, as shown in Figure 3B. Representative images of immune cell infiltration are presented in Figure 4F, illustrating that the combination treatment markedly increased the infiltration of CD11c+ and CD3+ cells (Figure 4G). In addition, tumor relapse after Ad‐E7P vaccination highlights the potential of NK cell mediated anti‐tumor immunity in tumors with deficient or reduced MHC class I expression. To this end, we analyzed NK cell infiltration in tumor tissues from different treatment groups from experimental mice, as shown in Figure 3B. We noted that combination therapy induced a marked increase in NK cells (Figure S13), which promoted NK cell mediated cytotoxicity, contributed to tumor control, and provided an effective strategy to prevent immune evasion.

### 2.5 RNA Sequencing Uncovers the Differential Gene Expression Profiles in Tumors

To identify immune regulatory changes following treatment, we performed RNA sequencing (RNA‐seq) on TC‐1 tumors harvested on day 17 from a repeated experiment shown in Figure 3A. Principal component analysis (PCA) revealed distinct transcriptional profiles among the treatment groups, with the combination group clearly separated from both the Ad‐E7P and SKV‐012 groups, indicating that combination therapy induced significant transcriptomic changes (Figure 5A). The corresponding Venn diagram (Figure S14A) and the heatmaps of differentially expressed genes (Figures 5B and S14B) further illustrate the transcriptomic differences among the treatment groups. Volcano plot further revealed a set of immune‐related genes that were significantly upregulated after treatment, including Nkg7, cxcl12, cxcl10, and Ifit3 (Figures 5C and S15A). Gene set enrichment analysis (GSEA) also showed enrichment of pathways associated with the adaptive immune response, positive regulation of natural killer (NK) cell‐mediated cytotoxicity, and innate immune response (Figures 5D and S15B). Gene ontology (GO) enrichment chord revealed upregulation of IFNG, IL12A, GZMA, CD28, CD8B1, and IL21, genes involved in inflammatory responses and innate and adaptive immune activation (Figure 5E). Similarly, pathway enrichment analysis using KEGG identified significant enrichment in the NF‐kappa B signaling pathway, antigen processing and presentation, Th1 and Th2 cell differentiation, Th17 cell differentiation, natural killer cell‐mediated cytotoxicity, and chemokine signaling pathway (Figure S16), indicating that the treatment elicited broad immune activation at the transcriptomic level.

To further assess whether alterations in immune responses were associated with differential expression of transcription factors, we identified differentially expressed transcription factors and generated a Sankey plot to illustrate the target genes and their corresponding transcription factors and transcription factor families. We found that Il7r (a critical regulator of thymic development and T‐cell proliferation) was significantly upregulated across all three treatment groups. In addition, Il12a, carried by SKV‐012, was specifically upregulated in both the SKV‐012 and combination treatment groups (Figure 5F). Overall, our transcriptomic analysis characterized the immunological landscape after treatment, highlighting the distinct and potentially synergistic effects of SKV‐012 and Ad‐E7P.

### 2.6 Combination Therapy Promotes Memory T Cell Response, Provides Durable Protection Against TC‐1 Tumor Rechallenge, and Exhibits Antitumor Activity in mEERL Model

**FIGURE 6:**
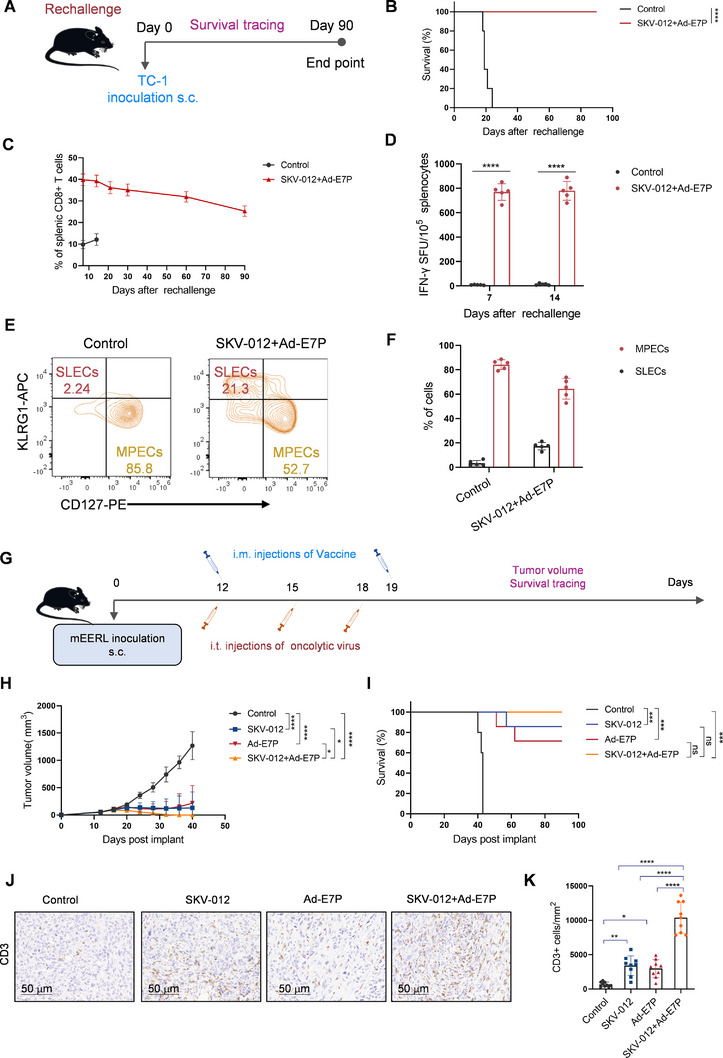
Combination of Ad‐E7P vaccine and SKV‐012 provides long‐term protection in the TC‐1 tumor model and induces robust antitumor immunity in the mEERL tumor model. (A) Experimental design. Mice cured by the SKV‐012+Ad‐E7P combination treatment were re‐inoculated with 1 × 10^6^ TC‐1 tumor cells in the left flank on Day 60, and survival was monitored. Untreated mice served as controls. (B) Survival kinetics of mice after tumor rechallenge (Control, *n*  =  5 mice; SKV‐012+Ad‐E7P, *n * =  10 mice). (C) On Days 7, 14, 21, 30, 60, and 90 post‐tumor challenge, the proportion of CD8+ T cells in the spleen was assessed by flow cytometry (*n* = 3 per group). (D) On Days 7 and 14 post‐tumor challenge, spleens were harvested, and the frequency of IFN‐γ‐producing T cells was assessed using an ELISPOT assay following in vitro stimulation with the E7 peptide (*n* = 5 per group). (E and F) Differential expression of KLRG1 and CD127 on spleen T cells. Representative contour plots (E, Day 7) and quantification (F) of KLRG1 and CD127 expression are shown (*n* = 5 per group). (G) Experimental Scheme. C57BL/6 mice were subcutaneously implanted with 2 × 10^6^ mEERL tumor cells. Mice were treated with Ad‐E7P, SKV‐012, Ad‐E7P + SKV‐012, or PBS (control) on Day 12, when tumor volumes reached approximately 50 mm^3^. For Ad‐E7P group, mice received two doses of 10^9^ Ad‐E7P administered once a week. For SKV‐012 group, mice received three intratumoral injections of 10^6^ PFU SKV‐012 every three days. For combination treatment group, mice received two doses of 10^9^ VP Ad‐E7P once a week and three doses of 10^6^ PFU SKV‐012 intratumorally every 3 days. Tumor volume was monitored. (H) Tumor growth curves in mEERL model (*n* = 5 per group). (I) Survival kinetics in mEERL model (Control, *n*  =  5 mice; Treatment group, *n*  =  7 mice). Mice from independent experimental cohorts. (J and K) Representative images of IHC staining for CD3 in mEERL tumor tissue sections on Day 20 (K), and quantification of CD3+ cells per tumor area (J) (cells/mm^2^) (*n* = 3 mice per group; *N* = 3 images/field of view per mouse).

Immune memory is considered a key factor in achieving long‐term tumor control. Except for the combination treatment group, which induced complete tumor regression (Figure 3B), both the vaccine and SKV‐012 as single agents show limited therapeutic potential, as shown in Figures 2E and 3B. To determine whether the combination treatment induce lasting memory immunity, we used a group of mice with regressing tumors from the repeated experiment of the combination treatment group (Figure 3B) and paired them with age‐matched, treatment‐naïve mice for rechallenge (Figure 6A). Significantly, compared to the control group, these mice were still protected against rechallenge with TC‐1 tumors, suggesting that Ad‐E7P+SKV‐012 induces sustained immunity (Figure 6B). Additionally, long‐term immune monitoring indicated a higher frequency of CD8+ T cells in the Ad‐E7P+SKV‐012 group (Figure 6C). ELISpot assays revealed that splenocytes from the rechallenged mice exhibited a stronger response to the epitopes (Figure 6D). We observed a shift from memory precursor effector cells (MPECs, KLRG1^−^ CD127^+^) to short‐lived effector cells (SLECs, KLRG1^+^ CD127^−^) on Day 7 after rechallenge (Figure 6E,F). In addition, memory T cells (Figure S17A), including antigen‐specific memory T‐cell populations (Figure S17B), were generated and distributed across both effector memory (Tem) and central memory (Tcm) subsets. These data demonstrate the functionality of combination therapy‐primed immune memory and highlight memory T cells as rapid responders upon antigen re‐exposure.

We further evaluated the antitumor efficacy of SKV‐012 and Ad‐E7P inmEERL tumor model, another widely used model for HPV‐associated malignancies (Figure 6G). Mice were euthanized on Day 40. All treatments effectively suppressed tumor growth and prolonged survival compared to the control; however, complete tumor regression was observed only in the combination treatment group (Figure 6H). Despite this, survival analysis revealed no significant differences among the three treatment groups (Figure 6I). IHC staining revealed that the combination therapy induced a higher infiltration of CD3+ T cells compared to the monotherapy groups in mEERL tumor tissues (Figure 6J,K). These findings collectively underscore the therapeutic potential of the SKV‐012 and Ad‐E7P combination in HPV‐associated tumors.

### 2.7 Vaccine Enhances Antitumor Efficacy With SKV‐012 in Vitro

**FIGURE 7:**
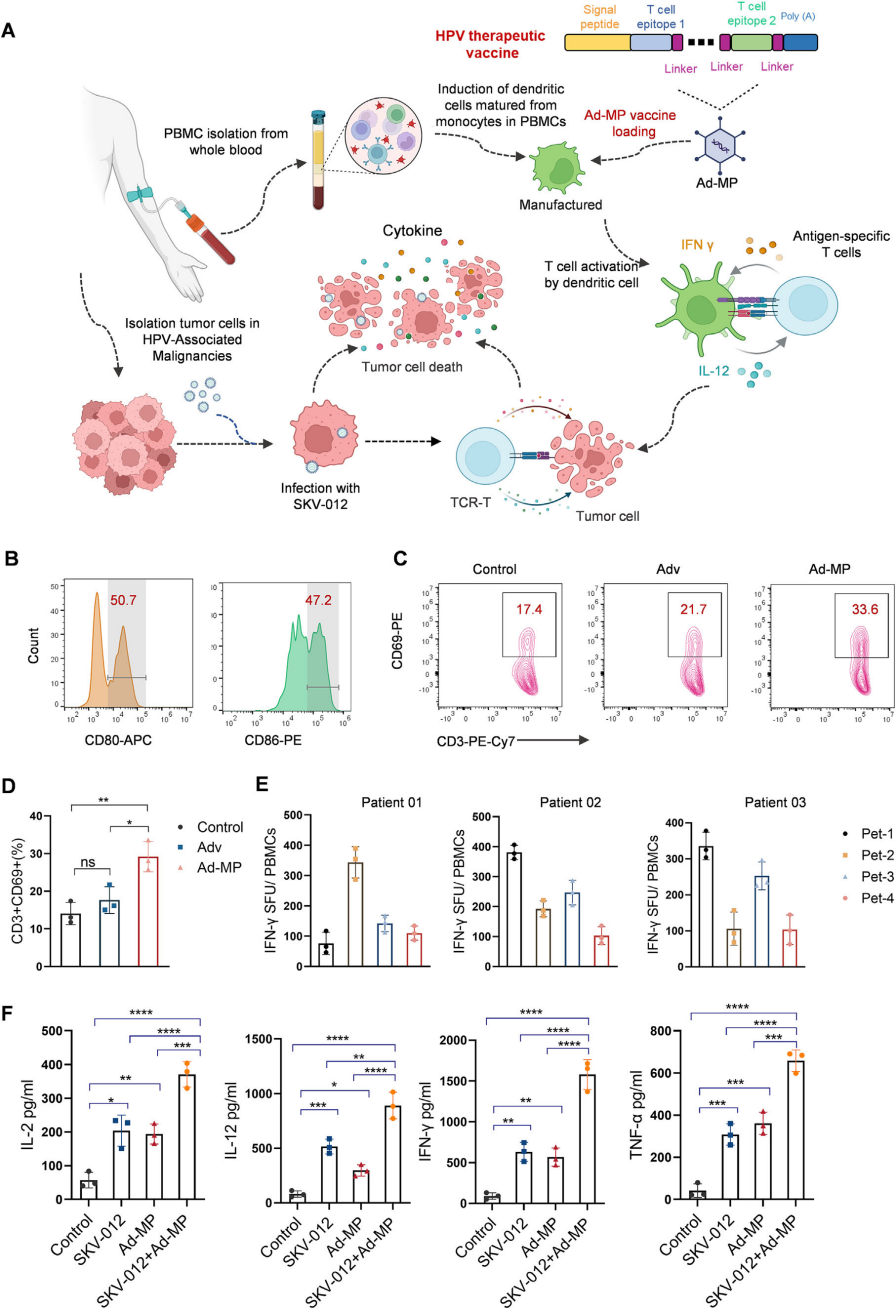
Ad‐MP triggers antigen‐specific T cell response and enhances antitumor efficacy with SKV‐012 in vitro. (A) Experimental design for assessing the SKV‐012 + Ad‐E7P immune response in vitro. The figure panel was created in BioRender. (B) Representative flow cytometry plots of CD80 and CD86 expression in DCs. DCs were isolated and induced from the peripheral blood of HPV‐related tumor patients and loaded with the Ad‐MP vaccine, and were tracked at Day7. (C and D) Representative flow cytometry plots of activated CD69 expression in T cells (C) and quantification of CD69+CD3+ T cells are shown (D). The Ad‐MP‐loaded DCs were co‐cultured with autologous PBMCs at a ratio of 1:100 for 24 h (*n* = 3 per group). Empty‐loaded DC were prepared as a control. (E) The number of IFN‐γSFUin PBMCs was assessed after 24 h of stimulation with single peptides (*n* = 3 per group). Before performing the ELISPOT assay, antigen‐loaded DCs were pre‐co‐cultured with autologous PBMCs from HPV‐related tumor patients for 24 h (*n* = 3 per group). (F) The concentrations of IL‐12, IFN‐γ, IL‐2, and TNF‐α in the supernatants were measured by ELISA after 48 h of co‐culture of primary tumor cells with autologous PBMCs and DCs. Prior to co‐culturing with activated autologous PBMCs, tumor cells were infected with SKV‐012 at an MOI of 0.01 for 24 h to assess responses (*n* = 3 per group). Data are presented as mean ± SD. One‐way ANOVA followed by Tukey's multiple comparisons test was used for statistical analysis. (*p < 0.05, **p < 0.01, ***p < 0.001, ****p < 0.0001). *ns*, not significant.

In our previous studies, a Phase 1 clinical trial (NCT06080984) involving SKV‐012 was initiated for patients with advanced solid tumors in China [27]. We observed that patients with advanced disease and substantial tumor burden poorly responded to single‐agent regimens, resulting in rapid disease progression. In this study, we demonstrated that the combination of oncolytic virus and the Ad‐E7P vaccine elicited a potent antitumor response in HPV‐related TC‐1 and mEERL mouse models, as shown in Figures 1, 2, 3, 4, 5, 6. Hence, we developed a candidate vaccine for humans, Ad‐MP, which includes HLA‐recognized E6/E7 epitopes [28, 29], and further investigated the response of this vaccine and oncolytic virus in advanced HPV‐related tumors in vitro (Figure 7A).

We prepared monocyte‐derived dendritic cells (Mo‐DCs) using granulocyte macrophage colony‐stimulating factor (GM‐CSF) and IL‐4 (described in Supporting Information) [30]. These DCs demonstrated increased expression levels of the costimulatory molecules CD80 and CD86 (Figure 7B) and exhibited typical dendritic morphology upon loading with Ad‐MP by (Figure S18). Increased accumulation of tumor antigens within DCs is essential for effective antigen presentation, serves as a key step for DC‐T cell crosstalk and the activation of antigen‐specific T cells. Following Ad‐MP loading onto DCs, we observed a marked upregulation of CD69 expression in CD3+ T cells during co‐culture with autologous PBMCs for 24 h (Figure 7C,D). We further evaluated patient‐specific responses to individual peptides included in the Ad‑MP vaccine using ELISPOT assays with autologous PBMCs. Following stimulation with single peptides, we observed an increase in IFN‐γ production (Figure 7E), but the response varied among patients, reflecting differences in HLA epitope recognition. We isolated primary tumor cells from Patient 03 and assessed cytokine production in the co‐culture supernatants after 48 h using ELISA. We observed increased production of IL‐12, IFN‐γ, IL‐2, and TNF‐α, particularly in the combination treatment group.

The findings suggest that the Adv‐based multi‐epitope vaccine, which can effectively initiate antigen‐specific immune responses, may further enhance antitumor efficacy through dual synergistic mechanisms when combined with the oncolytic virus SKV‐012, supporting its potential for future clinical applications.

## 3 Discussion

Vaccines represent an emerging treatment modality for tumors, with recent significant advancements in clinical applications and regulatory approvals [31, 32, 33], Despite the therapeutic benefits are being developed for advanced HPV‐associated tumors [34, 35], the clinical benefit of vaccine monotherapy is limited. In this study, we developed two therapeutic vaccine candidates, Ad‐E7P and Ad‐MP, based on MHC molecule polymorphisms and the distinct peptide epitopes recognized in mice and humans, and evaluated their potential in combination with an oncolytic virus SKV‐012 for HPV‐related tumors. Our findings demonstrate that this combinational strategy generates potent therapeutic effects and prevents tumor recurrence by inducing synergistic immune responses at both systemic and local levels, providing mechanistic proof of concept for patients with advanced HPV‐associated tumors.

Although MHC‐restricted T‐cell epitopes in E6 and E7 have been identified for most HPV‐associated tumors, antigen‐specific T‐cell reactivity is generally limited [36, 37, 38]. The potential advantage of epitope‐based peptide vaccines lies in the accumulation of antigens on the surface of antigen‐presenting cells (APCs), which facilitates the delivery of cognate T cell help to prime cytotoxic T lymphocytes (CTLs). In these scenarios, our vaccines were fused with lysosomal signal peptide and protease cleavage sites to optimize antigen presentation and enhance immunogenicity.

Several therapeutic vaccines targeting HPV‐related malignancies have been developed based on mRNA or adenovirus platforms and have shown efficacy in animal models. Recent publications indicate that adenovirus‐based vaccine candidates induced strong antigen‐specific T cell responses 14 days after vaccination, with antibody titers continuing to increase until day 28 [39, 40]. Our observations with Ad‐E7P showed that the vaccine elicited robust CD8+ T cell responses and significantly inhibited tumor growth after two doses, even in the absence of adjuvant, highlighting the potential of Ad‐based vaccines in immunotherapy. While MHC II restricted epitopes can induce CD4 T cell responses, the precise restriction has not been fully assessed due to the Ad‐E7P vaccine induces a predominantly CD8 T cell response. Our flow cytometry data further confirmed that CD8+ T cells constituted a substantial portion of the T cell response in lymph nodes, spleens, and tumors. Additionally, while B cell contributions have been described following Ad‐based vaccine administration [41, 42], the quality of the antibody responses has not been determined. Furthermore, it is noteworthy that the immune response in vaccinated animals after rechallenge was largely unchanged compared with the pre‐rechallenge response, probably due to the pre‐existing immunity in vaccinated animals, which induced a rapid recall T‐cell response upon antigen re‐exposure. The mechanism of this recall response is not well studied, although the formation of memory T cells, a fundamental feature of adaptive immunity, has been reported.

Patients with advanced or recurrent HPV‐positive cancer exhibit a highly suppressed TME [43, 44, 45], which limits the efficacy of therapeutic vaccination. To further attenuate the immunosuppressive TME and enhance the antitumor effects of the vaccine, we designed a combination treatment with an IL‐12‐loaded oncolytic virus. The therapeutic potential and safety of IL‐12 armed oncolytic viruses have been widely validated in both preclinical and clinical studies [46, 47]. Local delivery of IL‐12 via in situ injection of OVs, as applied in our study, minimizes toxicities while providing potent antitumor efficacy. We found that the IL‐12‐loaded oncolytic virus enhanced direct tumor cell killing, promoted dendritic cell (DC) antigen presentation, and triggered adaptive immunity, leading to durable antitumor responses. Previous studies have shown that oncolytic virus can induce epitope spreading of tumor antigens and elicit diverse tumor‐specific T cell responses. Given that TC‐1 mouse tumors commonly express HPV oncoproteins E6 and E7, the availability of detectable antigens and neoantigens is limited. Investigating the potential of combination therapy with OV to induce epitope spreading in other tumor models is warranted. Surprisingly, we found that compared with the Ad‐E7P group, the SKV‐012 group upregulated the H2‐M2 gene encoding MHC class I glycoprotein (Figure S14A), which may offset the downregulation of MHC‐I after vaccine immunization. We also observed a positive regulation of natural killer (NK) cells in the combination therapy (Figure S13). These complex interactions may collectively reverse immune escape.

While our studies provide important insights into the development of HPV vaccines and combination therapies, they also have some limitations that should be addressed in future investigations. First, although Ad platforms have been widely used as vaccine candidates due to their established safety and efficacy, the widespread pre‐existing immunity to the Ad5 serotype in humans remains a major challenge, restricting their clinical application and repeated use. To extend vaccine applicability, chimeric Ad5 vectors incorporating fiber proteins from other serotypes (e.g., Ad3, Ad11, and Ad35), and vectors derived from other species (such as chimpanzee‐derived ChAd6, ChAd7, and ChAd68), have been developed to improve transduction efficiency and overcome pre‐existing immunity. Therefore, the development of new vaccine strategies, particularly mRNA‐based platforms with safety, efficacy, and rapid production, is highly desirable to overcome these limitations. It would be interesting to investigate whether the discrepancy in protective efficacy and combination effects varies with the vaccine platform. Second, we used a low dose of the oncolytic virus to explore its synergistic effects with the vaccine as previously described, but the maximum tolerated dose for optimal vaccine–virus synergy warrants further investigation. Third, accumulating evidence indicates that multiple immunosuppressive mechanisms within the tumor microenvironment (TME) restrict the efficacy of immunotherapy. However, our study mainly focused on immune‐enhancing cell populations, whereas immunosuppressive subsets such as regulatory T cells (Tregs), M2 macrophages, and myeloid‐derived suppressor cells (MDSCs), which are known to regulate the immunosuppressive TME and promote treatment resistance, were not thoroughly examined. Among these populations, Tregs suppress immune responses by secreting inhibitory cytokines such as interleukin‐10 (IL‐10) and transforming growth factor‐β (TGF‐β), inducing DC dysfunction through the downregulation of CD80/CD86 expression, and mediating suppression via cytolysis and metabolic disruption [48]. M2 macrophages are also critical regulators that limit antitumor immunity by producing immunosuppressive factors such as IL‐10 and TGF‐β, which in turn support other suppressive cell populations including MDSCs and Tregs [49]. In addition, TME factors such as hypoxia and acidic pH conditions further compromise DC physiology, thereby preventing T cell activation and T cell‐mediated immunity. Thus, using oncolytic viruses to reprogram immunosuppressive TAMs holds great potential for boosting vaccine efficacy. Finally, while our findings are incredibly promising in animal models, how the observed immunogenicity and protection translate to human cancers remains unknown. Humanized patient‐derived xenograft (PDX) models provide a valuable platform to investigate immune‐tumor interactions, which will be critical for evaluating the efficacy of vaccines and combination therapies in humans. Employing a large, late‐stage tumor model to assess the antitumor immune response of the vaccine or combination therapy may more accurately simulate the immunosuppressive microenvironment characteristic of advanced tumors.

Collectively, our data provide a detailed evaluation of an Ad‐based vaccine in combination with oncolytic virus therapy in preclinical models of HPV‐induced tumors. Our findings point to an intricate mechanism involving crosstalk between local and systemic immune responses, which enhances the efficacy of immunotherapy and contributes to durable tumor control. Future studies will build on these observations to explore the clinical potential of this approach for advanced HPV‐associated malignancies.

## 4 Materials and methods

### 4.1 Vaccine Vector Generation

The AdMax strategy was used to generate the Ad‐E7P vaccine vector, as previously described [50]. The sequence encoding the concatemeric peptide epitopes was as follows: signal peptide (SP)‐antigen1‐linker‐antigen2‐…‐linker‐poly A. Both the SP sequence, derived from an endosomal or lysosomal protein, and the linker sequences, which incorporate protease cleavage sites, are designed to optimize the processing and presentation of antigens by antigen‐presenting cells (APCs), as described in US Patent Application 20190008938. The amino acid sequence of the vaccines mentioned in Table S2.

The coding regions were codon optimized and synthesized by GenScript (Nanjing, China). It was then subcloned into the shuttle plasmid pDC516 using EcoRI and HindIII restriction enzymes (New England Biolabs). Subsequently, the shuttle plasmid containing the epitope expression cassette and the adenovirus genomic plasmid pBHgloxΔE1, E3 were co‐transfected into 293 cells to generate the recombinant adenoviral vectors Ad‐E7P and Ad‐MP. Empty adenoviral vector was used as controls.

### 4.2 Cell Lines

Lin KY et al. described that TC‐1 tumor cells were derived from primary lung epithelial cells of C57BL/6 mice through the transformation with genes encoding c‐Ha‐ras and HPV‐16 E6 and E7 [51]. These cells were cultured in RPMI‐1640 (Gibco), containing 10% fetal bovine serum (FBS) (Gibco), and 1% penicillin‐streptomycin (PS) and maintained at 37°C with 5% CO_2_ in the incubator. mEERL (mouse E6/E7/hRas/luciferase) cell line was purchased from Merck (Cat#SCC627) and cultured in DMEM/F12 (Gibco), supplemented with 10% FBS. Vero and 293 cell lines were purchased from ATCC and cultured in complete cell medium supplemented with 10% FBS and 100 U/mL penicillin‐streptomycin. All cell lines used in this study have undergone authentication by short tandem repeat (STR) analysis to confirm their identity and have been tested mycoplasma‐free.

### 4.3 Animals

Six‐ to eight‐week‐old female C57BL/6 mice were purchased from GemPharmatech (Nanjing, China) and maintained under specific pathogen‐free conditions. All animal experiments were conducted in compliance with the guidance of the Institutional Animal Care and Use Committee of Sichuan University (Chengdu, China). The experimental details are described in the corresponding figure legends.

### 4.4 IFN‐γ ELISPOT Assays

IFN‐γ production in mice was assessed using an enzyme‐linked immunospot (ELISPOT) assay following the manufacturer's protocol (DAKEWE, Cat#DKW22‐2000‐096). Splenocytes were harvested after immunization and seeded at 1 × 10^5^ cells per well in a pre‐coated PVDF plate, then incubated with the E7 peptide (RAHYNIVTF; GenScript) at a concentration of 10 µg/mL for 24 h at 37°C.

The frequency of IFN‐γ–producing T cells in human PBMCs was assessed by ELISpot following co‐culture with mature DCs at a DC‐to‐lymphocyte ratio of 1:100 for 24 h. Subsequently, PBMCs were seeded at 2 × 10^5^ cells per well in ELISpot plates (human IFN‐γ ELISpot kit, R&D Systems, Cat#EL285) and stimulated with single peptide 1–4 at a concentration of 10 µg/mL for 24 h at 37°C. At the end of incubation, the ELISpot assay was carried out in accordance with the manufacturer's instructions. Spots were visualized and enumerated using an Immunospot analyzer (Cellular Technology Limited) or an AID ELISpot reader (Autoimmun Diagnostika GmbH). Data are presented as spot‐forming units (SFU) per well.

### 4.5 Flow Cytometry Staining

Antibodies used for flow cytometry are listed in Table S3. Single‐cell suspensions from spleens or lymph nodes were blocked with anti‐CD16/32 Fc‐receptor blockade (BioLegend) for 30 min. Subsequently, the cells were stained extracellularly to assess the populations of DCs, cDCs, pDCs, T cells, B cells, Tcm, Tem, SLECs, and MPECs. For tumor microenvironment analysis, TC‐1 tumors were digested into single‐cell suspensions by Collagenase type IV (Sigma–Aldrich) and DNase‐I (Roche) as previously described [52], and then stained with live/dead dye in PBS for 20 min. Samples were subsequently washed with PBS and blocked with anti‐CD16/32 Fc‐receptor blockade (BioLegend) for 30 min. Then applicable surface‐staining antibodies were added. Immune cell phenotypes were characterized by pre‐gating on singlets and viable cells and determined as follows (see the Supporting Information for gating strategies): DCs (CD45^+^CD11b^+^CD11c^+^MHC II^+^), macrophage (CD45^+^CD11b^+^F4/80^+^), T cells (CD45^+^CD3^+^), CD8+ T cells (CD45^+^CD3^+^CD8^+^), CD4+ T cells (CD45^+^CD3^+^CD4^+^), MDSCs (CD45^+^CD11b^+^Gr‐1^+^), PMN‐MDSCs (CD45+CD11b+Gr‐1+Ly6G^+^Ly6C^low^), and M‐MDSCs (CD45^+^CD11b^+^Gr‐1^+^CD11b^+^Ly6G^−^Ly6C^high^).

### 4.6 RNA Sequencing

Total RNA samples from tumor tissues of TC‐1 experimental mice were used for RNA‐seq analysis, performed by Shanghai OE Biotech.

### 4.7 Statistical Analysis

Statistical analyses were performed using GraphPad software. Two‐tailed unpaired Student's *t* tests were conducted to compare the differences between two experimental groups. Multiple comparisons involving more than two groups were analyzed using one‐ or two‐way ANOVA with Tukey's or Bonferroni's correction. Survival curves were assessed using the log‐rank (Mantel‐Cox) test. Data are presented as means ± SD. A *p*‐value of less than 0.05 was considered statistically significant: *p < 0.05, **p < 0.01, ***p < 0.001, ****p < 0.0001; ns, not significant.

## Author Contributions

**Conceptualization**: A.T., H.Y. and N.Y. **Data curation**: N.Y, L.X., M.Z. and Z.Z. **Formal analysis**: N.Y, L.X., M.Z. and H.L. **Funding acquisition**: A.T. and H.Y. **Investigation**: N.Y, L.X., M.Z., Z.Z. and H.L. **Methodology**: N.Y, L.X., M.Z., Z.Z., H.L., Y.C., Z.Z. and W.Z. **Validation**: N.Y., L.X., M.Z., H.L., Y.C. and Z.Z. **Visualization**: N.Y, L.X., M.Z. and W.Z. **Resources**: W.Z, Z.W., H.L, J.L., Z.J., P.Z., G.W., H.X. and H.Y. **Supervision**: A.T., H.Y. and Z.Z. **Writing – original draft**: N.Y, L.X. and M.Z. **Writing – review and editing**: all authors. All authors have read and approved the final manuscript.

## Funding

This work was supported by funding from the National Key Research and Development Program of China (2023YFC3403303 & 2023YFC3403304), the National Natural Science Foundation of China (32471551 & 82401342), and the Frontiers Medical Center, Tianfu Jincheng Laboratory Foundation (TFJC2023010006), the Natural Science Foundation of Sichuan Province of China (2026NSFSC1913), the Postdoctor Research Fund of West China Hospital, Sichuan University (2024HXBH177).

## Ethics Approval

All animal experiments were conducted in accordance with the guidelines approved by the Medical Ethics Committee of Hospital of West China Hospital of Sichuan University Biomedical Ethics Committee (20210535A). All procedures involving human participants were approved by the Institutional Review Board of West China Hospital (2023‐833) for the collection of specimens. Informed consent was obtained from all participants involved in the study.

## Conflicts of Interest

The authors declare no conflicts of interest.

## Supporting information




**Supporting Table 1**: List of peptides used.
**Supporting Table 2**: Amino acid sequence of the vaccines.
**Supporting Table 3**: Flow cytometry antibodies used in this work.
**Supporting Figure 1**: Gating strategy to quantify DC subsets and activation status.
**Supporting Figure 2**: Gating strategy for spleens to quantify B cells, T cells, CD8+ T cells, E7‐specific CD8 + T cells, and IFN γ+ CD8+ T cells. The IFN γ‐FMO control serves to accurately identify the positive population.
**Supporting Figure 3**: Gating strategy to quantify SLECs and MPEC subsets in CD8+ T cells.
**Supporting Figure 4**: Gating strategy to quantify MDSC, PMN‐MDSC and M‐MDSC subsets.
**Supporting Figure 5**: Gating strategy for TC‐1 tumors to determine immune cell infiltration.
**Supporting Figure 6**: Gating strategy for TC‐1 tumors to determine E7‐specific CD8 + T cells. The E7‐Tetramer FMO control serves to accurately identify the positive population.
**Supporting Figure 7**: Identification of E7‐specific T cell responses using tetramer staining. (A and B) Gating strategy for identifying E7‐specific CD8+ T cells in lymph nodes and spleens after vaccination (A), and quantification of E7‐specific CD8+^+^ T cells (B). (C and D) Gating strategy for TC‐1 tumors (C) and quantification of E7‐specific CD8+ T cells (D). E7‐tetramer FMO control was used to identify the positive population. Data are presented as the means ± SD. One‐way analysis of variance (ANOVA) with Tukey's multiple comparisons test was performed for all comparisons (*p < 0.05, **p < 0.01, ***p < 0.001, ****p < 0.0001). *ns*, not significant.
**Supporting Figure 8**: Tertiary lymphoid structures in tumor tissues. Representative images of tertiary lymphoid structures detected in formalin‐fixed paraffin‐embedded TC‐1 tumor sections by hematoxylin and eosin (H&E) staining (left) or by immunohistochemistry staining showing CD3+ T‐cell zones, CD20+ B‐cell zones, and CD21+ follicular dendritic cell (FDC) zones. Scale bars: 200 µm (overview) and 50 µm (zoomed‐in view).
**Supporting Figure 9**: Decreased MHC‐I and E7 expression contributes to TC‐1 tumor relapse after vaccination. (A and B) Representative images of immunofluorescence (A) and IHC (B) staining of paraffin‐embedded sections of TC‐1 tumor tissue at pre‐ (Day 21 after the primary inoculation) and post‐rechallenge (Day 21 post‐rechallenge). (C) Representative western blot image and quantitative analysis of E7 expression in TC‐1 tumor tissue at pre‐ and post‐rechallenge. (D) Representative flow cytometry plots and quantification of E7‐specific CD8+ T cells in spleens on day 7 after rechallenge. Data are presented as the means ± SD. Two‐tailed *t*‐test was used for statistical analysis (*p < 0.05, **p < 0.01, ***p < 0.001, ****p < 0.0001). *ns*, not significant.
**Supporting Figure 10**: Characteristics of dendritic cells mature in lymph nodes and spleen. (A) Representative flow cytometric analysis of CD40 expression on dendritic cells in lymph nodes (left), and quantification of mean fluorescence intensity (MFI) of CD40 expression (left) (*n* = 3 per group). (B) Representative flow cytometric analysis of CD86 expression on dendritic cells in spleens (Right). (*p < 0.05, **p < 0.01, ***p < 0.001, ****p < 0.0001). *ns*, not significant. Data are presented as the means ± SD. One‐way analysis of variance (ANOVA) with Tukey's multiple comparisons test was performed for all comparisons (*p < 0.05, **p < 0.01, ***p < 0.001, ****p < 0.0001). *ns*, not significant.
**Supporting Figure 11**: Tetramer staining of E7‐specific cells in spleens. (A) Representative flow cytometry plots are shown for E7‐specific CD8+ T cells in spleens. (B) Quantification of E7‐specific CD8+ T cells using tetramer‐based flow cytometry (*n* = 3 per group). (*p < 0.05, **p < 0.01, ***p < 0.001, ****p < 0.0001). *ns*, not significant. Data are presented as the means ± SD. One‐way analysis of variance (ANOVA) with Tukey's multiple comparisons test was performed for all comparisons (*p < 0.05, **p < 0.01, ***p < 0.001, ****p < 0.0001). *ns*, not significant.
**Supporting Figure 12**: Vaccine dose‐dependent synergy with oncolytic virus. (A and B) Tumor growth curves (*n* = 5 per group). C57BL/6 mice were subcutaneously inoculated with 10^6^ TC‐1 tumor cells in the right flank. When tumor volumes reached approximately 50–100 mm^3^, mice were received two reduced vaccine doses (1E+08 and 5E+08, respectively) of vaccine once a week and/or three doses of 10^6^ PFU SKV‐012 every two days. Mice were euthanized on days 23–25, as tumor volumes in the 1E+08 group approached the ethical endpoint of 1500 mm^3^.
**Supporting Figure 13**: NK cell infiltration in TC‐1 tumors. Representative IHC staining images and corresponding quantification of NK1.1 in TC‐1 tumor tissue after treatment (*n* = 3 per group). Scale bars, 50 µm. (*p < 0.05, **p < 0.01, ***p < 0.001, ****p < 0.0001). *ns*, not significant. Data are presented as the means ± SD. One‐way analysis of variance (ANOVA) with Tukey's multiple comparisons test was performed for all comparisons (*p < 0.05, **p < 0.01, ***p < 0.001, ****p < 0.0001). *ns*, not significant.
**Supporting Figure 14**: Differentially expressed genes in RNA‐seq data. (A and B) Venn diagram (A) and heatmap (B) of differentially expressed genes (DEGs) identified from RNA‐seq analysis.
**Supporting Figure 15**: Volcano plot and GSEA analysis of transcriptomic changes. (A) Volcano plot showing differentially expressed genes between the two groups. Green dots represent downregulated genes and purple dots represent upregulated genes. (B) GSEA revealing enrichment of pathways related to the adaptive immune response, positive regulation of NK cell–mediated cytotoxicity, and innate immune activation after treatment.
**Supporting Figure 16**: KEGG pathway enrichment bubble chart shows the top 20 enriched pathways (FDR < 0.05).
**Supporting Figure 17**: Evaluation of memory T‐cell response. (A and B) Representative dot plots showing total memory T cells (A, left) and antigen‐specific memory CD8+ T cells (B, left) in splenic T cells at day 7 post–tumor challenge. Effector memory (Tem, CD44^high^CD62L^−^) and central memory (Tcm, CD44^high^CD62L^+^) subsets are indicated, and the corresponding quantification of Tem and Tcm populations in the spleen is shown on the right (A, *n* = 5 per group; B, *n* = 3 per group). Data are presented as the means ± SD. Two‐tailed *t*‐test was used for statistical analysis (*p < 0.05, **p < 0.01, ***p < 0.001, ****p < 0.0001). *ns*, not significant.
**Supporting Figure 18**: DCs loaded with Ad‐MP. Microscopic image of Mo‐DCs after Ad‐MP loading. Scale bar, 100 µm.

## Data Availability

All data associated with this study are in the paper or Supporting Information. The raw sequence data reported in this paper have been deposited in the Genome Sequence Archive (Genomics, Proteomics & Bioinformatics 2025) in National Genomics Data Center (Nucleic Acids Res 2025), China National Center for Bioinformation/Beijing Institute of Genomics, Chinese Academy of Sciences (GSA: CRA032507) that are publicly accessible at https://ngdc.cncb.ac.cn/gsa.

## References

[1] A. A. Shamseddine , B. Burman , N. Y. Lee , D. Zamarin , and N. Riaz , “Tumor Immunity and Immunotherapy for HPV‐Related Cancers,” Cancer Discovery 11, no. 8 (2021): 1896–1912. 33990345 10.1158/2159-8290.CD-20-1760 PMC8338882

[2] F. Wei , D. Georges , I. Man , I. Baussano , and G. M. Clifford , “Causal Attribution of human Papillomavirus Genotypes to Invasive Cervical Cancer Worldwide: A Systematic Analysis of the Global Literature,” Lancet (London, England) 404, no. 10451 (2024): 435–444. 39097395 10.1016/S0140-6736(24)01097-3

[3] R. L. Ferris and W. Westra , “Oropharyngeal Carcinoma With a Special Focus on HPV‐Related Squamous Cell Carcinoma,” Annual Review of Pathology 18 (2023): 515–535. 10.1146/annurev-pathmechdis-031521-041424 PMC11227657 36693202

[4] M. Falcaro , A. Castañon , B. Ndlela , et al., “The Effects of the National HPV Vaccination Programme in England, UK, on Cervical Cancer and Grade 3 Cervical Intraepithelial Neoplasia Incidence: A Register‐based Observational Study,” Lancet (London, England) 398, no. 10316 (2021): 2084–2092. 34741816 10.1016/S0140-6736(21)02178-4

[5] M. Saraiya , E. R. Unger , T. D. Thompson , et al., “US Assessment of HPV Types in Cancers: Implications for Current and 9‐valent HPV Vaccines,” Journal of the National Cancer Institute 107, no. 6 (2015): djv086. 25925419 10.1093/jnci/djv086 PMC4838063

[6] C. L. Trimble , M. P. Morrow , K. A. Kraynyak , et al., “Safety, Efficacy, and Immunogenicity of VGX‐3100, a Therapeutic Synthetic DNA Vaccine Targeting human Papillomavirus 16 and 18 E6 and E7 Proteins for Cervical Intraepithelial Neoplasia 2/3: A Randomised, Double‐blind, Placebo‐controlled Phase 2b Trial,” Lancet (London, England) 386, no. 10008 (2015): 2078–2088. 26386540 10.1016/S0140-6736(15)00239-1 PMC4888059

[7] M. L. Bagarazzi , J. Yan , M. P. Morrow , et al., “Immunotherapy Against HPV16/18 Generates Potent TH1 and Cytotoxic Cellular Immune Responses,” Science Translational Medicine 4, no. 155 (2012): 155ra138. 10.1126/scitranslmed.3004414 PMC4317299 23052295

[8] J. Sadoff , G. Gray , A. Vandebosch , et al., “Final Analysis of Efficacy and Safety of Single‐Dose Ad26.COV2.S,” The New England Journal of Medicine 386, no. 9 (2022): 847–860. 35139271 10.1056/NEJMoa2117608 PMC8849184

[9] L. Coughlan , E. J. Kremer , and D. M. Shayakhmetov , “Adenovirus‐based Vaccines‐a Platform for Pandemic Preparedness Against Emerging Viral Pathogens,” Molecular Therapy: The Journal of the American Society of Gene Therapy 30, no. 5 (2022): 1822–1849. 35092844 10.1016/j.ymthe.2022.01.034 PMC8801892

[10] D. van Riel and E. de Wit , “Next‐generation Vaccine Platforms for COVID‐19,” Nature Materials 19, no. 8 (2020): 810–812. 32704139 10.1038/s41563-020-0746-0

[11] E. Borcoman , A. Lalanne , J. P. Delord , et al., “Phase Ib/II Trial of Tipapkinogene Sovacivec, a Therapeutic human papillomavirus16‐vaccine, in Combination With avelumab in Patients With Advanced human papillomavirus16‐positive Cancers,” European Journal of Cancer (Oxford, England: 1990) 191 (2023): 112981. 37506588 10.1016/j.ejca.2023.112981

[12] T. C. van der Sluis , F. J. van Haften , S. van Duikeren , et al., “Delayed Vaccine‐induced CD8(+) T Cell Expansion by Topoisomerase I Inhibition Mediates Enhanced CD70‐dependent Tumor Eradication,” Journal for Immunotherapy of Cancer 11, no. 11 (2023): e007158. 38030302 10.1136/jitc-2023-007158 PMC10689370

[13] M. Weller , N. Butowski , D. D. Tran , et al., “Rindopepimut With Temozolomide for Patients With Newly Diagnosed, EGFRvIII‐expressing Glioblastoma (ACT IV): A Randomised, Double‐blind, International Phase 3 Trial,” The Lancet Oncology 18, no. 10 (2017): 1373–1385. 28844499 10.1016/S1470-2045(17)30517-X

[14] E. A. Mittendorf , B. Lu , M. Melisko , et al., “Efficacy and Safety Analysis of Nelipepimut‐S Vaccine to Prevent Breast Cancer Recurrence: A Randomized, Multicenter, Phase III Clinical Trial,” Clinical Cancer Research: An Official Journal of the American Association for Cancer Research 25, no. 14 (2019): 4248–4254. 31036542 10.1158/1078-0432.CCR-18-2867

[15] R. Ramlau , E. Quoix , J. Rolski , et al., “A Phase II Study of Tg4010 (Mva‐Muc1‐Il2) in Association With Chemotherapy in Patients With Stage III/IV Non‐small Cell Lung Cancer,” Journal of Thoracic Oncology: Official Publication of the International Association for the Study of Lung Cancer 3, no. 7 (2008): 735–744. 18594319 10.1097/JTO.0b013e31817c6b4f

[16] S. Meulewaeter , I. Aernout , J. Deprez , et al., “Alpha‐galactosylceramide Improves the Potency of mRNA LNP Vaccines Against Cancer and Intracellular Bacteria,” Journal of Controlled Release: Official Journal of the Controlled Release Society 370 (2024): 379–391. 38697317 10.1016/j.jconrel.2024.04.052

[17] S. Kreiter , M. Diken , A. Selmi , et al., “FLT3 ligand Enhances the Cancer Therapeutic Potency of Naked RNA Vaccines,” Cancer Research 71, no. 19 (2011): 6132–6142. 21816907 10.1158/0008-5472.CAN-11-0291

[18] R. Fu , R. Qi , H. Xiong , et al., “Combination Therapy With Oncolytic Virus and T Cells or mRNA Vaccine Amplifies Antitumor Effects,” Signal Transduction and Targeted Therapy 9, no. 1 (2024): 118. 38702343 10.1038/s41392-024-01824-1 PMC11068743

[19] K. DePeaux and G. M. Delgoffe , “Integrating Innate and Adaptive Immunity in Oncolytic Virus Therapy,” Trends in Cancer 10, no. 2 (2024): 135–146. 37880008 10.1016/j.trecan.2023.09.012 PMC10922271

[20] H. L. Kaufman , F. J. Kohlhapp , and A. Zloza , “Oncolytic Viruses: A New Class of Immunotherapy Drugs,” Nature Reviews Drug Discovery 14, no. 9 (2015): 642–662. 26323545 10.1038/nrd4663 PMC7097180

[21] R. Li , P. H. Shah , T. F. Stewart , et al., “Oncolytic Adenoviral Therapy plus Pembrolizumab in BCG‐unresponsive Non‐muscle‐invasive Bladder Cancer: The Phase 2 CORE‐001 Trial,” Nature Medicine 30, no. 8 (2024): 2216–2223. 10.1038/s41591-024-03025-3 38844794

[22] K. Harrington , D. J. Freeman , B. Kelly , J. Harper , and J. C. Soria , “Optimizing Oncolytic Virotherapy in Cancer Treatment,” Nature Reviews Drug Discovery 18, no. 9 (2019): 689–706. 31292532 10.1038/s41573-019-0029-0

[23] M. Liu , S. Hu , N. Yan , K. D. Popowski , and K. Cheng , “Inhalable Extracellular Vesicle Delivery of IL‐12 mRNA to Treat Lung Cancer and Promote Systemic Immunity,” Nature Nanotechnology 19, no. 4 (2024): 565–575. 10.1038/s41565-023-01580-3 PMC12930425 38212521

[24] B. Brook , V. Duval , S. Barman , et al., “Adjuvantation of a SARS‐CoV‐2 mRNA Vaccine With Controlled Tissue‐specific Expression of an mRNA Encoding IL‐12p70,” Science Translational Medicine 16, no. 757 (2024): eadm8451. 39047117 10.1126/scitranslmed.adm8451

[25] G. Trinchieri , “Interleukin‐12 and the Regulation of Innate Resistance and Adaptive Immunity,” Nature Reviews Immunology 3, no. 2 (2003): 133–146. 10.1038/nri1001 12563297

[26] S. Bevers , S. A. A. Kooijmans , E. Van de Velde , et al., “mRNA‐LNP Vaccines Tuned for Systemic Immunization Induce Strong Antitumor Immunity by Engaging Splenic Immune Cells,” Molecular Therapy 30, no. 9 (2022): 3078–3094. 35821637 10.1016/j.ymthe.2022.07.007 PMC9273295

[27] Z. Jiang , N. Yang , and J. Jin , “Preclinical and Clinical Evaluation of Intratumoral Injection of an IL‐12 Expressing SKV‐012 Oncolytic Virus for Advanced Solid Tumors,” Journal for Immunotherapy of Cancer 13, no. 6 (2025): e011642. 40484644 10.1136/jitc-2025-011642 PMC12161328

[28] L. M. Draper , M. L. Kwong , A. Gros , et al., “Targeting of HPV‐16+ Epithelial Cancer Cells by TCR Gene Engineered T Cells Directed Against E6,” Clinical Cancer Research: An Official Journal of the American Association for Cancer Research 21, no. 19 (2015): 4431–4439. 26429982 10.1158/1078-0432.CCR-14-3341 PMC4603283

[29] N. B. Nagarsheth , S. M. Norberg , A. L. Sinkoe , et al., “TCR‐engineered T Cells Targeting E7 for Patients With Metastatic HPV‐associated Epithelial Cancers,” Nature Medicine 27, no. 3 (2021): 419–425. 10.1038/s41591-020-01225-1 PMC9620481 33558725

[30] M. Hiasa , M. Abe , A. Nakano , et al., “GM‐CSF and IL‐4 Induce Dendritic Cell Differentiation and Disrupt Osteoclastogenesis Through M‐CSF Receptor Shedding by Up‐regulation of TNF‐alpha Converting Enzyme (TACE),” Blood 114, no. 20 (2009): 4517–4526. 19762488 10.1182/blood-2009-04-215020

[31] J. S. W. Borgers , D. Lenkala , V. Kohler , et al., “Personalized, Autologous Neoantigen‐specific T Cell Therapy in Metastatic Melanoma: A Phase 1 Trial,” Nature Medicine 31, no. 3 (2025): 881–893. 10.1038/s41591-024-03418-4 PMC11922764 39753970

[32] D. A. Braun , G. Moranzoni , V. Chea , et al., “A Neoantigen Vaccine Generates Antitumour Immunity in Renal Cell Carcinoma,” Nature 639, no. 8054 (2025): 474–482. 39910301 10.1038/s41586-024-08507-5 PMC11903305

[33] P. D. Katsikis , K. J. Ishii , and C. Schliehe , “Challenges in Developing Personalized Neoantigen Cancer Vaccines,” Nature Reviews Immunology 24, no. 3 (2024): 213–227. 10.1038/s41577-023-00937-y PMC12001822 37783860

[34] M. J. Welters , T. C. van der Sluis , H. van Meir , et al., “Vaccination During Myeloid Cell Depletion by Cancer Chemotherapy Fosters Robust T Cell Responses,” Science Translational Medicine 8, no. 334 (2016): 334ra52. 10.1126/scitranslmed.aad8307 27075626

[35] L. Maldonado , J. E. Teague , M. P. Morrow , et al., “Intramuscular Therapeutic Vaccination Targeting HPV16 Induces T Cell Responses That Localize in Mucosal Lesions,” Science Translational Medicine 6, no. 221 (2014): 221ra13. 10.1126/scitranslmed.3007323 PMC4086631 24477000

[36] M. J. Atherton , K. B. Stephenson , J. K. Nikota , et al., “Preclinical Development of Peptide Vaccination Combined With Oncolytic MG1‐E6E7 for HPV‐associated Cancer,” Vaccine 36, no. 16 (2018): 2181–2192. 29544689 10.1016/j.vaccine.2018.02.070

[37] C. S. Eberhardt , H. T. Kissick , M. R. Patel , et al., “Functional HPV‐specific PD‐1(+) Stem‐Like CD8 T Cells in Head and Neck Cancer,” Nature 597, no. 7875 (2021): 279–284. 34471285 10.1038/s41586-021-03862-z PMC10201342

[38] S. M. Norberg and C. S. Hinrichs , “Engineered T Cell Therapy for Viral and Non‐viral Epithelial Cancers,” Cancer Cell 41, no. 1 (2023): 58–69. 36400016 10.1016/j.ccell.2022.10.016 PMC9839504

[39] S. Lanini , S. Capone , A. Antinori , et al., “GRAd‐COV2, a Gorilla adenovirus‐based Candidate Vaccine Against COVID‐19, Is Safe and Immunogenic in Younger and Older Adults,” Science Translational Medicine 14, no. 627 (2022): eabj1996. 34698501 10.1126/scitranslmed.abj1996

[40] C. Dold , L. Marsay , N. Wang , et al., “An Adenoviral‐vectored Vaccine Confers Seroprotection Against Capsular Group B Meningococcal Disease,” Science Translational Medicine 15, no. 701 (2023): eade3901. 37343082 10.1126/scitranslmed.ade3901

[41] E. J. Kremer , “Pros and Cons of Adenovirus‐Based SARS‐CoV‐2 Vaccines,” Molecular Therapy: The Journal of the American Society of Gene Therapy 28, no. 11 (2020): 2303–2304. 33065038 10.1016/j.ymthe.2020.10.002 PMC7546260

[42] S. Capone , F. M. Fusco , S. Milleri , et al., “GRAd‐COV2 Vaccine Provides Potent and Durable Humoral and Cellular Immunity to SARS‐CoV‐2 in Randomized Placebo‐controlled Phase 2 Trial,” Cell Reports Medicine 4, no. 6 (2023): 101084. 37315558 10.1016/j.xcrm.2023.101084 PMC10243192

[43] M. Lechner , J. Liu , L. Masterson , and T. R. Fenton , “HPV‐associated Oropharyngeal Cancer: Epidemiology, Molecular Biology and Clinical Management,” Nature Reviews Clinical Oncology 19, no. 5 (2022): 306–327. 10.1038/s41571-022-00603-7 PMC8805140 35105976

[44] A. T. Ruffin , H. Li , L. Vujanovic , D. P. Zandberg , R. L. Ferris , and T. C. Bruno , “Improving Head and Neck Cancer Therapies by Immunomodulation of the Tumour Microenvironment,” Nature Reviews Cancer 23, no. 3 (2023): 173–188. 36456755 10.1038/s41568-022-00531-9 PMC9992112

[45] J. W. Youn , S. Y. Hur , J. W. Woo , et al., “Pembrolizumab plus GX‐188E Therapeutic DNA Vaccine in Patients With HPV‐16‐positive or HPV‐18‐positive Advanced Cervical Cancer: Interim Results of a Single‐arm, Phase 2 Trial,” Lancet Oncology 21, no. 12 (2020): 1653–1660. 33271094 10.1016/S1470-2045(20)30486-1

[46] K. Ye , F. Li , R. Wang , et al., “An Armed Oncolytic Virus Enhances the Efficacy of Tumor‐infiltrating Lymphocyte Therapy by Converting Tumors to Artificial Antigen‐presenting Cells in Situ,” Molecular Therapy: The Journal of the American Society of Gene Therapy 30, no. 12 (2022): 3658–3676. 35715953 10.1016/j.ymthe.2022.06.010 PMC9734027

[47] P. K. Bommareddy , H. Wakimoto , R. L. Martuza , H. L. Kaufman , S. D. Rabkin , and D. Saha , “Oncolytic herpes Simplex Virus Expressing IL‐2 Controls Glioblastoma Growth and Improves Survival,” Journal for Immunotherapy of Cancer 12, no. 4 (2024): e008880. 38599661 10.1136/jitc-2024-008880 PMC11015300

[48] D. A. Vignali , L. W. Collison , and C. J. Workman , “How Regulatory T Cells Work,” Nature Reviews Immunology 8, no. 7 (2008): 523–532. 10.1038/nri2343 PMC2665249 18566595

[49] A. Mantovani , P. Allavena , F. Marchesi , and C. Garlanda , “Macrophages as Tools and Targets in Cancer Therapy,” Nature Reviews Drug Discovery 21, no. 11 (2022): 799–820. 35974096 10.1038/s41573-022-00520-5 PMC9380983

[50] G. N. Medina , T. de Los Santos , and F. Díaz‐San Segundo , “Generation of Replication Deficient Human Adenovirus 5 (Ad5) Vectored FMD Vaccines,” Methods in Molecular Biology (Clifton, NJ) 2465 (2022): 155–175. 10.1007/978-1-0716-2168-4_9 35118621

[51] K. Y. Lin , F. G. Guarnieri , and K. F. Staveley‐O'Carroll , “Treatment of Established Tumors With a Novel Vaccine That Enhances Major Histocompatibility Class II Presentation of Tumor Antigen,” Cancer Research 56, no. 1 (1996): 21–26. 8548765

[52] Z. Zhang , N. Yang , H. Lu , et al., “Improved Antitumor Effects Elicited by an Oncolytic HSV‐1 Expressing a Novel B7H3nb/CD3 BsAb,” Cancer Letters 588 (2024): 216760. 38428724 10.1016/j.canlet.2024.216760

