# Supplementary material for: Combination of Vaccine With IL‐12‐Armed Oncolytic Virus SKV‐012 Synergistically Potentiates Immune Responses in HPV‐Associated Malignancies
Source: MedComm (2020). 2026 Apr 13;7(4):e70737. doi: 10.1002/mco2.70737 (PMC13077211; doi:10.1002/mco2.70737)
Supplement: Supplementary file 1 — Supporting Table 1: List of peptides used. Supporting Table 2: Amino acid sequence of the vaccines. Supporting Table 3: Flow cytometry antibodies used in this work. Supporting Figure 1: Gating strategy to quantify DC subsets and activation status. Supporting Figure 2: Gating strategy for spleens to quantify B cells, T cells, CD8+ T cells, E7‐specific CD8 + T cells, and IFN γ+ CD8+ T cells. The IFN γ‐FMO control serves to accurately identify the positive population. Supporting Figure 3: Gating strategy to quantify SLECs and MPEC subsets in CD8+ T cells. Supporting Figure 4: Gating strategy to quantify MDSC, PMN‐MDSC and M‐MDSC subsets. Supporting Figure 5: Gating strategy for TC‐1 tumors to determine immune cell infiltration. Supporting Figure 6: Gating strategy for TC‐1 tumors to determine E7‐specific CD8 + T cells. The E7‐Tetramer FMO control serves to accurately identify the positive population. Supporting Figure 7: Identification of E7‐specific T cell responses using tetramer staining. (A and B) Gating strategy for identifying E7‐specific CD8+ T cells in lymph nodes and spleens after vaccination (A), and quantification of E7‐specific CD8++ T cells (B). (C and D) Gating strategy for TC‐1 tumors (C) and quantification of E7‐specific CD8+ T cells (D). E7‐tetramer FMO control was used to identify the positive population. Data are presented as the means ± SD. One‐way analysis of variance (ANOVA) with Tukey's multiple comparisons test was performed for all comparisons (*p < 0.05, **p < 0.01, ***p < 0.001, ****p < 0.0001). ns, not significant. Supporting Figure 8: Tertiary lymphoid structures in tumor tissues. Representative images of tertiary lymphoid structures detected in formalin‐fixed paraffin‐embedded TC‐1 tumor sections by hematoxylin and eosin (H&E) staining (left) or by immunohistochemistry staining showing CD3+ T‐cell zones, CD20+ B‐cell zones, and CD21+ follicular dendritic cell (FDC) zones. Scale bars: 200 µm (overview) and 50 µm (zoomed‐in view). Supporting [file MCO2-7-e70737-s001.pdf]

Supplementary Information

Combination of vaccine with IL-12-armed oncolytic virus SKV-012 synergistically potentiates immune responses in HPV-associated malignancies

Nian Yang [1#], Long Xu [1#], Meijun Zheng[2#], Huaqing Lu[1], Yongdong Chen[1], Zhixiong Zhu[1], Wanqin Zeng[1], Zeng Wang[1], Hexian Li, Jia Li[1], Zheng Jiang[3], Pingfu Zeng[2], Guoqing Wang[4], Hai Xie[1], Zongliang Zhang[1*], Hui Yang[2*], Aiping Tong[1,5*]

[#]These authors contributed equally to the manuscript

[*]Correspondence: Dr Aiping Tong (aipingtong@scu.edu.cn), Dr Hui Yang (yh8806@163.com), and Dr. Zongliang Zhang (zhangzongliang2019@163.com)

[1]State Key Laboratory of Biotherapy and Cancer Center, Research Unit of Gene and Immunotherapy, Chinese Academy of Medical Sciences, Collaborative Innovation Center of Biotherapy, West China Hospital, Sichuan University; Chengdu 610041, Sichuan, China.

[2]Department of Otolaryngology- Head & Neck Surgery, West China Hospital, Sichuan University; Chengdu 610041, Sichuan, China.

[3]Department of Biotherapy, Cancer Center, West China Hospital, Sichuan University; Chengdu 610041, Sichuan, China.

[4]Department of Ophthalmology, West China Hospital, Sichuan University, West China Medical School, Chengdu 610041, Sichuan, China.

[5]Frontiers Medical Center, Tianfu Jincheng Laboratory, Chengdu 610212, Sichuan, China.

Supplementary materials and methods

Oncolytic virus SKV-012 manufacturing

The oncolytic viruses expressing murine or human IL-12, denoted as SKV-012, used in this study were constructed in our laboratory based on the YD06 strain. The generation of SKV-012 was described in the patent application CN 202510477383.X. SKV-012 was produced from infected Vero cells, harvested 72 hours post-infection, and purified. Then viral titers were determined using $TCID_{50}$ assays. The processing and storage of SKV-012 followed the preparation guidelines outlined in Appendix C of a prior publication[1].

Intracellular cytokine staining

Single-cell suspensions from lymph nodes or spleens were blocked with human TruStain FcX (BioLegend) and stained with anti-mouse CD3 and CD8 antibodies for 30 minutes, following the manufacturer's instructions. Subsequently, the cells were fixed and permeabilized using the BD Pharmingen Transcription Factor Buffer Set (BD Biosciences Pharmingen), then intracellularly stained with anti-mouse IFN-γ antibodies and analyzed by flow cytometry.

Flow cytometry was performed using an ID7000™ Spectral Cell Analyzer or a Beckman Coulter Cytoflex flow cytometer. FlowJo and CytExpert software were used for cytometry data analyses. Gating strategies are provided in supplemental information.

Tetramer generation and antigen-specific T cells detection

MHC monomers (Cat#H2M-M82E9-25μg), Streptavidin Protein-PE (Cat#STN-NP119-100μg), and Streptavidin Protein-FITC (Cat#STN-NF113-200ug) were purchased from Acro biosystems for tetramer generation and staining to assess antigen-specific responses. The approach involved

incubating biotinylated peptide–MHC monomers (pMHCs) with fluorochrome-conjugated streptavidin on ice for 30 minutes in the dark to generate tetramer mix.

Single-cell suspensions from spleens or tumors were blocked with TruStain FcX (BioLegend) and stained with anti-mouse CD3, CD8 antibodies, and tetramer at 37°C for 30 minutes and analyzed by flow cytometry.

Peptide synthesis

The antigen–specific peptide for in vitro immune response studies was synthesized from Sangon Biotech and dissolved in dimethyl sulfoxide (Sigma-Aldrich, D2650) for use. All peptide sequences are provided in Table S1.

IHC staining

Formalin-fixed, paraffin-embedded 5-μm tissue sections from experimental mice were stained with the following monoclonal antibodies: anti-mouse CD3ε (clone D7A6E, Cell Signaling Technology, Cat#85061), anti-mouse CD11c (clone D1V9Y, Cell Signaling Technology, Cat#97585), anti-mouse NK1.1 (clone E6Y9G, Cell Signaling Technology, Cat#39197T), anti-mouse CD20 (clone E3N7O, Cell Signaling Technology, Cat#70168), anti-CD21 (clone SC0681, HUABIO, Catalog#ET1610-61), and anti-E7 antibody (clone ED17, Santa Cruz Biotechnology, Cat#sc-6981). Enumeration of positive cells per square millimeter of tissue was performed using QuPath, and the mean density (cumulative optical density/area) was analyzed by ImageJ.

Immunofluorescence

For immunofluorescence, sections were stained with anti-mouse MHC Class I (E8E7N, Cell Signaling Technology, Cat#76828) rabbit monoclonal antibody (mAb) primary antibody, then

labeled with goat anti-rabbit fluorescent secondary antibody Alexa Fluor™ 594 (Invitrogen, Cat#A-11012) to detect MHC-I expression in the tumor tissues. Nuclei were counterstained with DAPI (Beyotime, Cat#C1005). Images were captured using a confocal microscope (Spin, OLYMPUS) and analyzed with ImageJ.

WB analyses

Tumor tissues were lysed in RIPA lysis buffer containing phosphatase and protease inhibitors (Beyotime). Equal protein amounts were separated by SDS-PAGE and analyzed by Western blot using an anti-E7 antibody (clone ED17, Santa Cruz Biotechnology, Cat# sc-6981), Anti-Beta Tubulin antibody (Proteintech, Cat# 10094-1-AP), HRP-conjugated goat anti-Rabbit (Proteintech, Cat# SA00001-2), and HRP-conjugated goat anti-Mouse (Proteintech, Cat# SA00001-1). Blots were imaged on an E-Blot Imager and analyzed using ImageJ.

Patient samples

For research (non-clinical) analyses, Patient-derived PBMCs were isolated from blood samples using density gradient centrifugation and then cryopreserved for downstream analyses or suspended in RPMI-1640 (Gibco) supplemented with 10% heat-inactivated highly defined FBS (Gibco).

Fresh tumor tissue was placed in separate tubes containing DMEM on ice. Tumor tissue was enzymatically and mechanically processed into 1-2 mm^3 fragments, followed by digestion into single-cell suspensions using Collagenase Type IV (Sigma-Aldrich) and DNase I (Roche) at 37°C for 30 minutes as previously described[2], and filtered through a 70 μm cell strainer (BD Falcon) Single-cell suspensions from tumor were then cultured in complete cell medium supplemented with 10% FBS and 100 U/mL penicillin-streptomycin for downstream applications.

Preparation of antigen-loaded DCs

Monocyte-derived DCs were prepared as described [3, 4]. Briefly, PBMCs were cultured for 7 days in RPMI-1640 (Gibco), supplemented with 10% heat-inactivated FBS (Gibco), granulocyte-macrophage colony-stimulating factor (GM-CSF; Gibco, Cat# PHC2015), and interleukin-4 (IL-4; Gibco, Cat# 200-04-20UG). Empty-loaded DC were prepared as a control. On day 7, immature DCs were stimulated with GM-CSF, IL4, and TNF-α (Gibco, Cat# 300-01A-50UG) and loaded with Ad-MP for 48 h to obtain mature DCs. Empty-loaded DC were prepared as a control. Approximately 50% of the cells were positive for CD80 and CD86 expression. These mature DCs were then harvested, counted and resuspended in cytokines-free RPMI-1640 medium for subsequent experiments.

Antigen reactive T-cell preparation

Mature DCs were harvested and cocultured with autologous PBMCs from HPV-related patients at a DC-to-lymphocyte ratio of 1:100 for 24 h. After coculturing, the cells were centrifuged, washed, and analyzed for the expression of the T cell activation marker CD69 by flow cytometry. Additionally, as described above, ELISPOT assays were performed to assess IFN-γ responses to single peptides.

References

1. Friedman GK, Johnston JM, Bag AK, et al. Oncolytic HSV-1 G207 Immunovirotherapy for Pediatric High-Grade Gliomas. *The New England journal of medicine*. 2021;384(17):1613-1622.

2. Zhang Z, Yang N, Lu H, et al. Improved antitumor effects elicited by an oncolytic HSV-1 expressing a novel B7H3nb/CD3 BsAb. *Cancer letters*. 2024;588:216760.

3. Perez CR, De Palma M. Engineering dendritic cell vaccines to improve cancer immunotherapy. *Nature communications*. 2019;10(1):5408.

4. Saxena M, Balan S, Roudko V, Bhardwaj N. Towards superior dendritic-cell vaccines for cancer therapy. *Nature biomedical engineering*. 2018;2(6):341-346.

Supplementary Tables

Supplementary Table 1. List of peptides used.

Vaccine	Target	Peptide	MHC
Ad-E7P	E7	RAHYNIVTF	H2-Db
Ad-MP	E7	YMLDLQPET (Pet-1)	HLA-A*02:01
		IVCPICSQK (Pet-2)	HLA-A*11:01
	E6	TIHDIILECV (Pet-3)	HLA-A*02:01
		TTLEQQYNK (Pet-4)	HLA-A*11:01

Supplementary Table 2. Amino acid sequence of the vaccines.

Vaccine	Sequence
Ad-E7P	MAAPGARRPLLLLLLAAGLAHGASARAHYNIVTFGFLGRAHYNIVTFTVGL RRAHYNIVTFRGRKRRSRAHYNIVTFKVSRRAHYNIVTFRGRKRRSRAHYNI VTFPMGLPRAHYNIVTFGGSGGS
Ad-MP	MAAPGSARRPLLLLLLLLLLGLMHCASAYMLDLQPETGFLGYMLDLQPETT VGLRYMLDLQPETKVSRIVCPICSQKRGRKRRSIVCPICSQKPMGLPIVCPICS QKKVSRTIHDIILECVGFLGTIHDIILECVKVSRTIHDIILECVRGRKRRSTTLEQ QYNKTVGLRTTLEQQYNKPMGAPTTLEQQYNKGGSGGS

Supplementary Table2. Flow cytometry antibodies used in this work.

ANTIBODIES/REAGENTS	MANUFACTURER	CATALOG NUMBER
ANTI-MOUSE CD16/32	BIOLEGEND	CAT#101320
ZOMBIE NIR^{TM}DYE	BIOLEGEND	LOT#B354830
ANTI-MOUSE CD45	BIOLEGEND	CAT#103132
ANTI-MOUSE CD3	BIOLEGEND	CAT#100237
ANTI-MOUSE CD8A	BIOLEGEND	CAT#100752
ANTI-MOUSE F4/80	BIOLEGEND	CAT#123116
ANTI-MOUSE CD11b	BIOLEGEND	CAT#101212
ANTI-MOUSE CD11c	BIOLEGEND	CAT#117338
ANTI-MOUSE CD86	BIOLEGEND	CAT#105008
ANTI-MOUSE LY6G	BD	CAT#560599
ANTI-MOUSE LY6C	BD	CAT560592
ANTI-MOUSE IFN-γ	BIOLEGEND	CAT#505826
ANTI-MOUSE CD127	BIOLEGEND	CAT#158204
ANTI-MOUSE KLRG-1	BIOLEGEND	CAT#138412
HUMAN TRUSTAIN FCX	BIOLEGEND	CAT# 422302
ANTI-HUMAN CD3	BIOLEGEND	CAT#300316
ANTI-HUMAN CD8	BIOLEGEND	CAT#300906
ANTI-HUMAN CD69	BIOLEGEND	CAT#985202
ANTI-HUMAN CD11C	BD	CAT#561356
ANTI-HUMAN CD80	BIOLEGEND	CAT#305219
ANTI-HUMAN CD86	BIOLEGEND	CAT#305405

Supplementary Figures

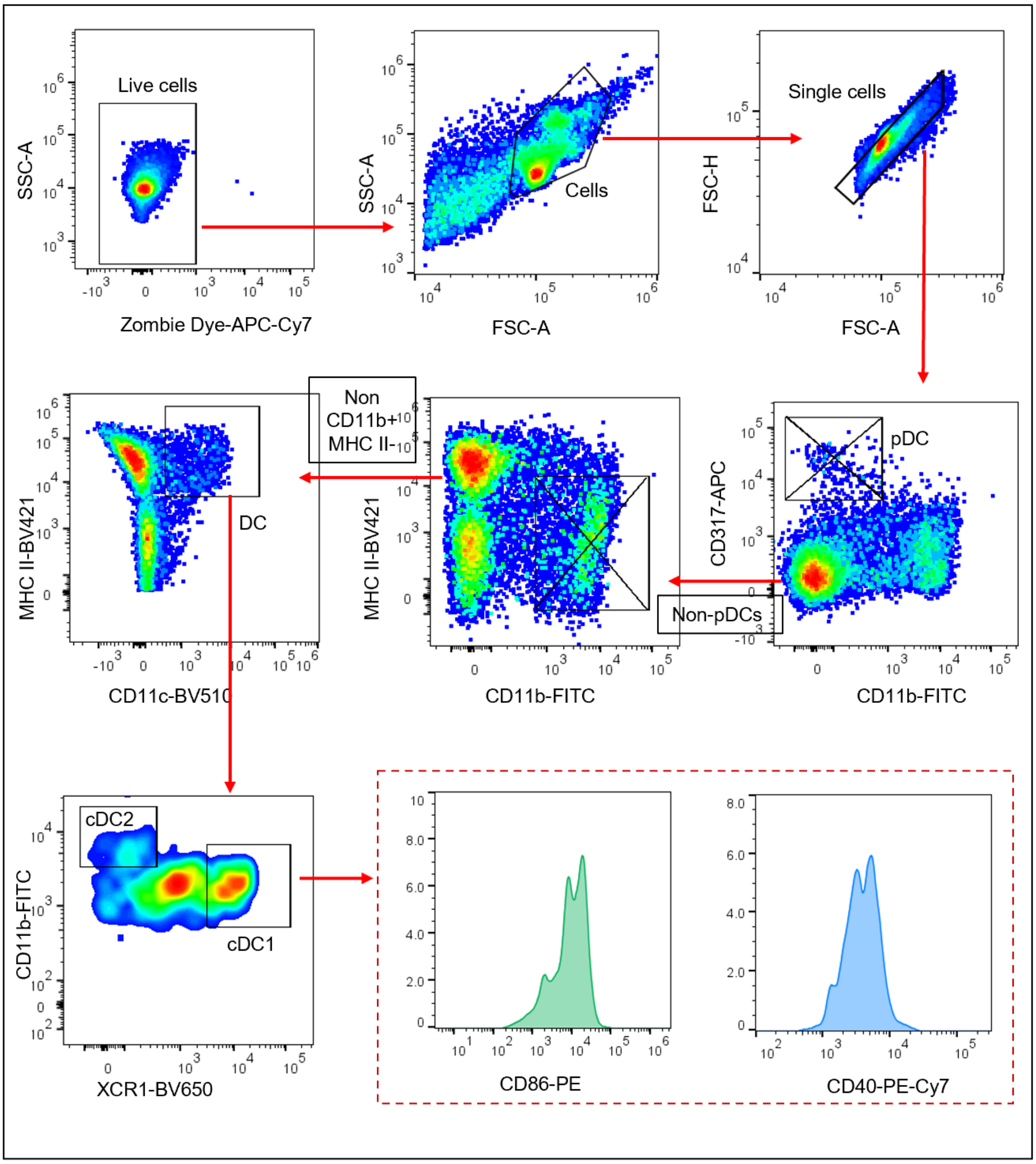

Figure S1. Gating strategy to quantify DC subsets and activation status.

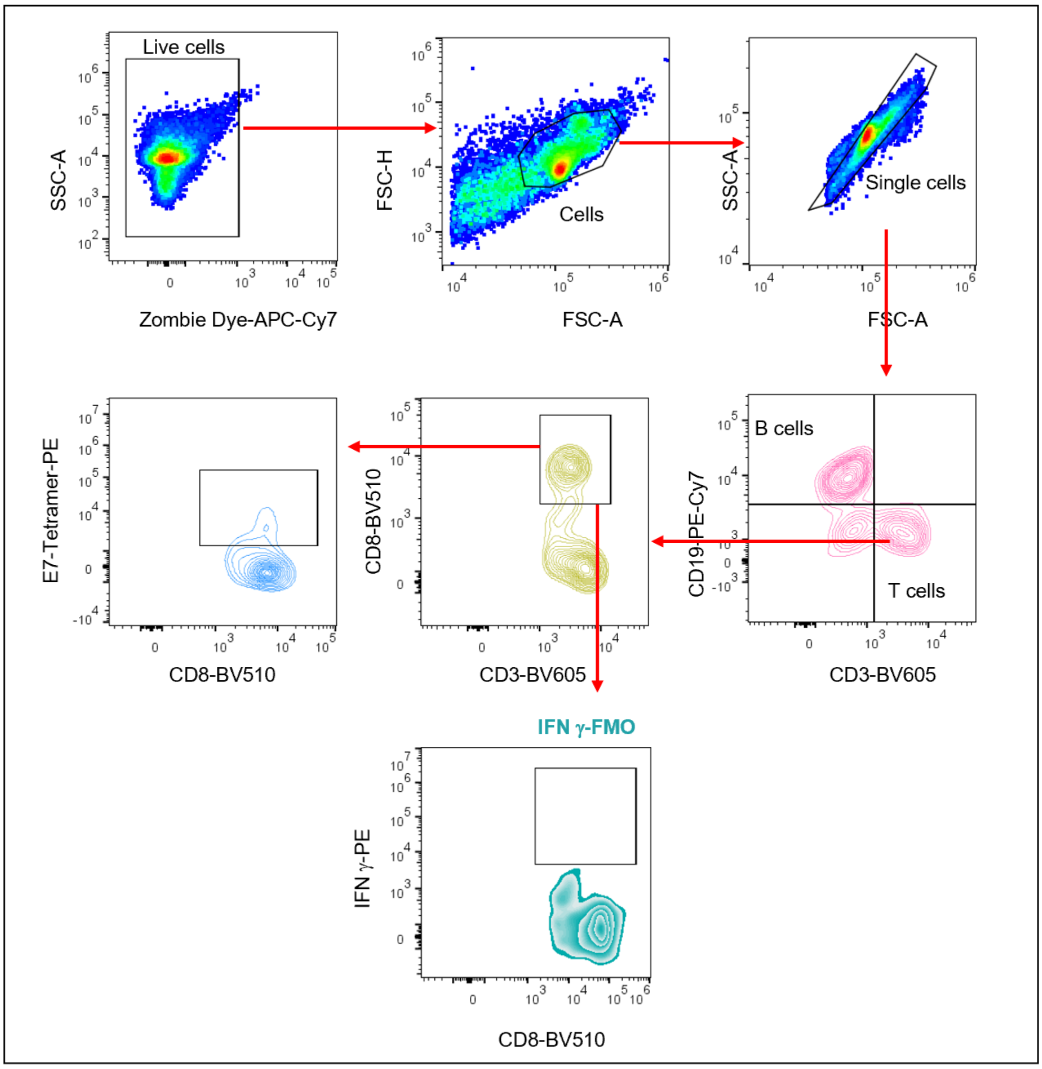

Figure S2. Gating strategy for spleens to quantify B cells, T cells, CD8+ T cells, E7-specific CD8 + T cells, and IFN γ+ CD8+ T cells. The IFN γ-FMO control serves to accurately identify the positive population.

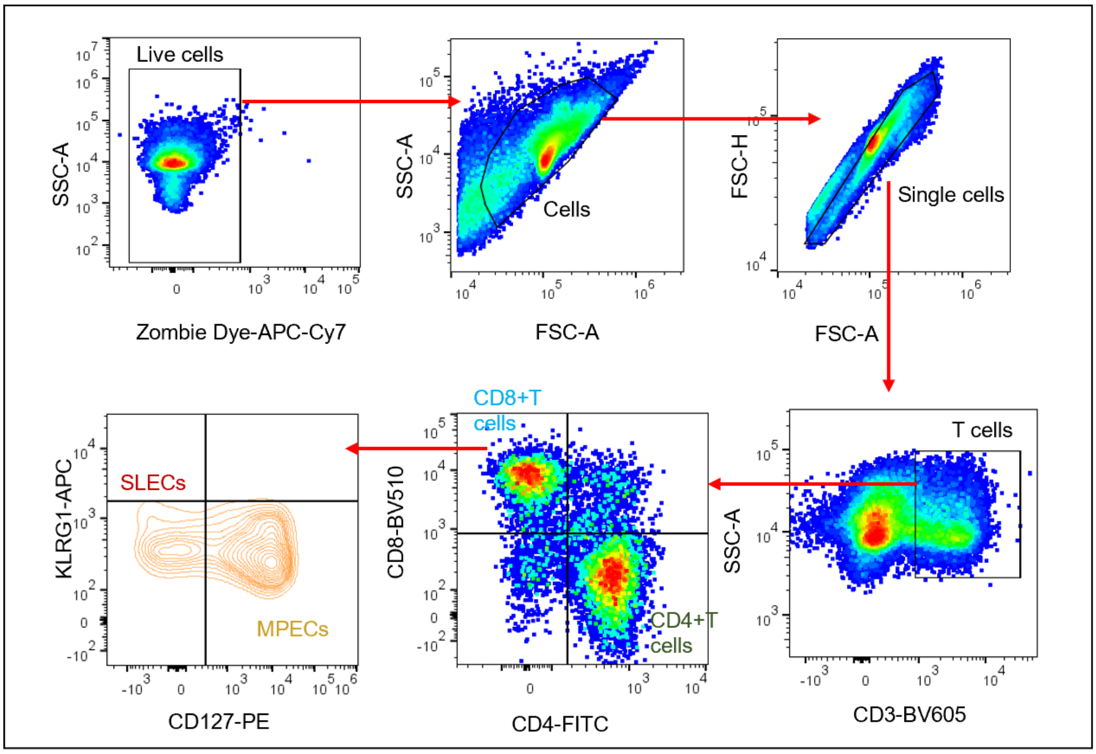

Figure S3. Gating strategy to quantify SLECs and MPEC subsets in CD8+ T cells.

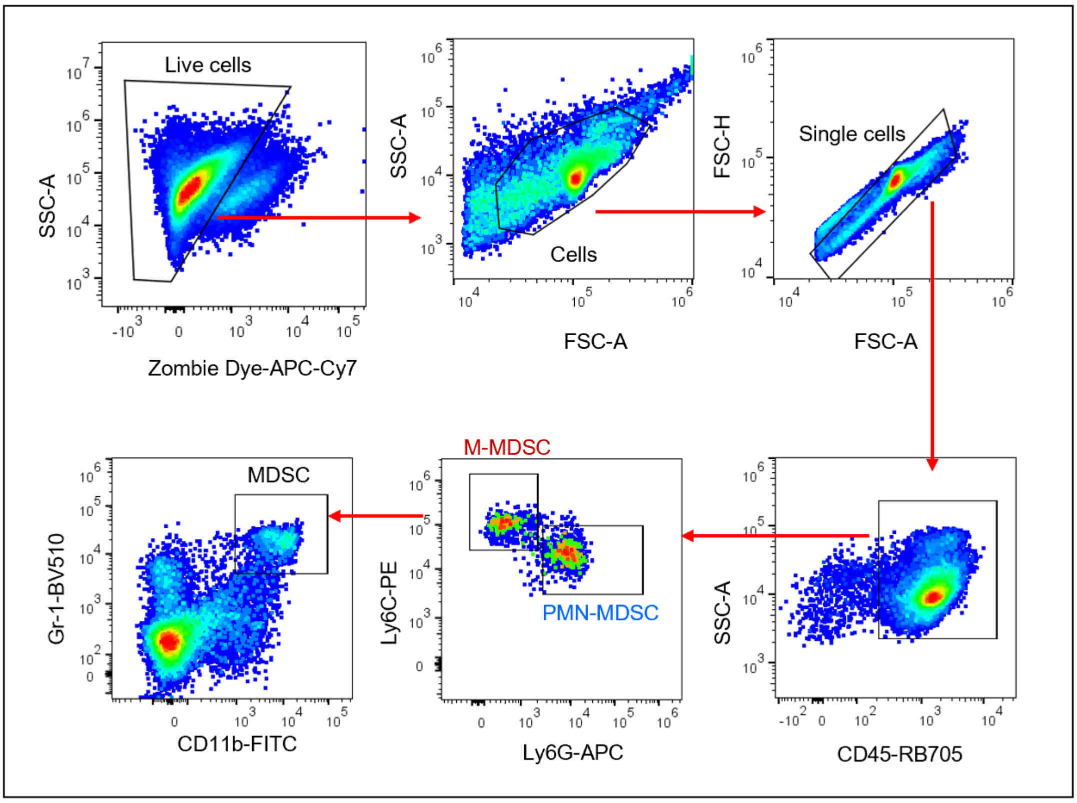

Figure S4. Gating strategy to quantify MDSC, PMN-MDSC and M-MDSC subsets

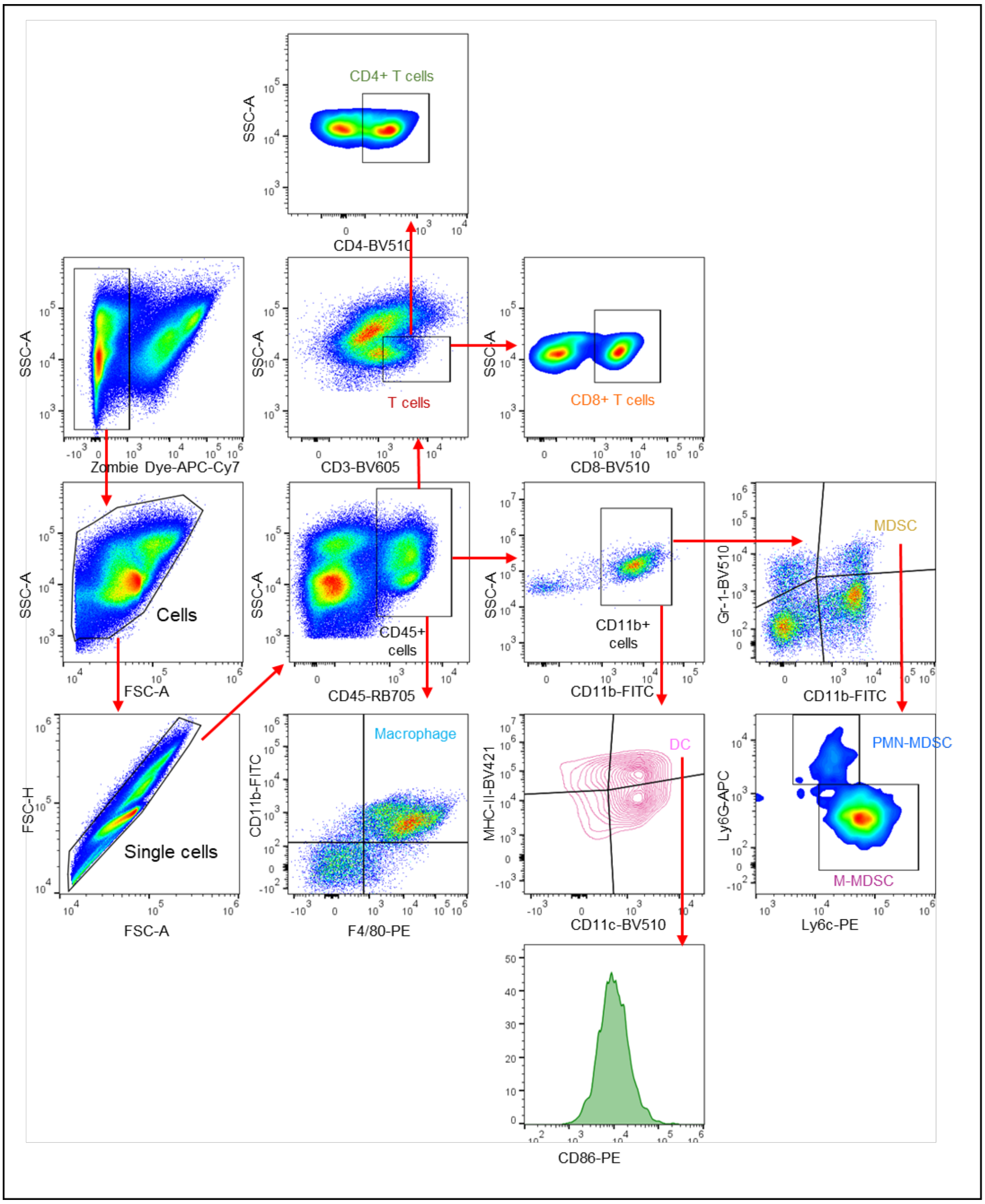

Figure S5. Gating strategy for TC-1 tumors to determine immune cell infiltration.

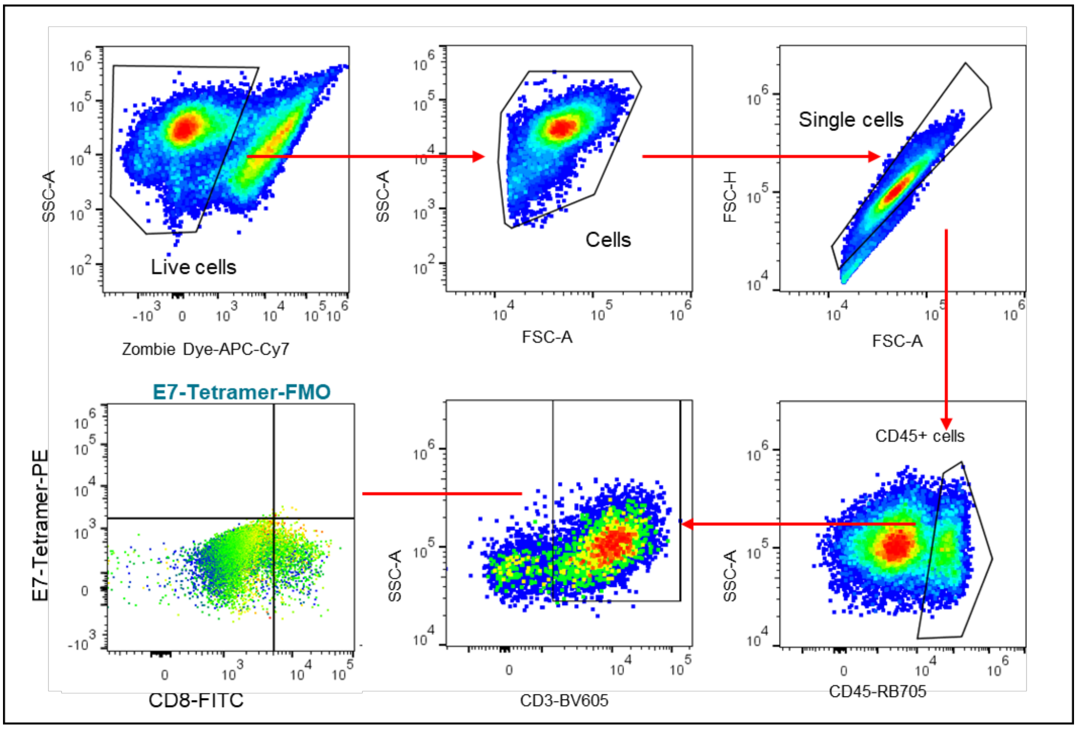

Figure S6. Gating strategy for TC-1 tumors to determine E7-specific CD8 + T cells. The E7-Tetramer FMO control serves to accurately identify the positive population.

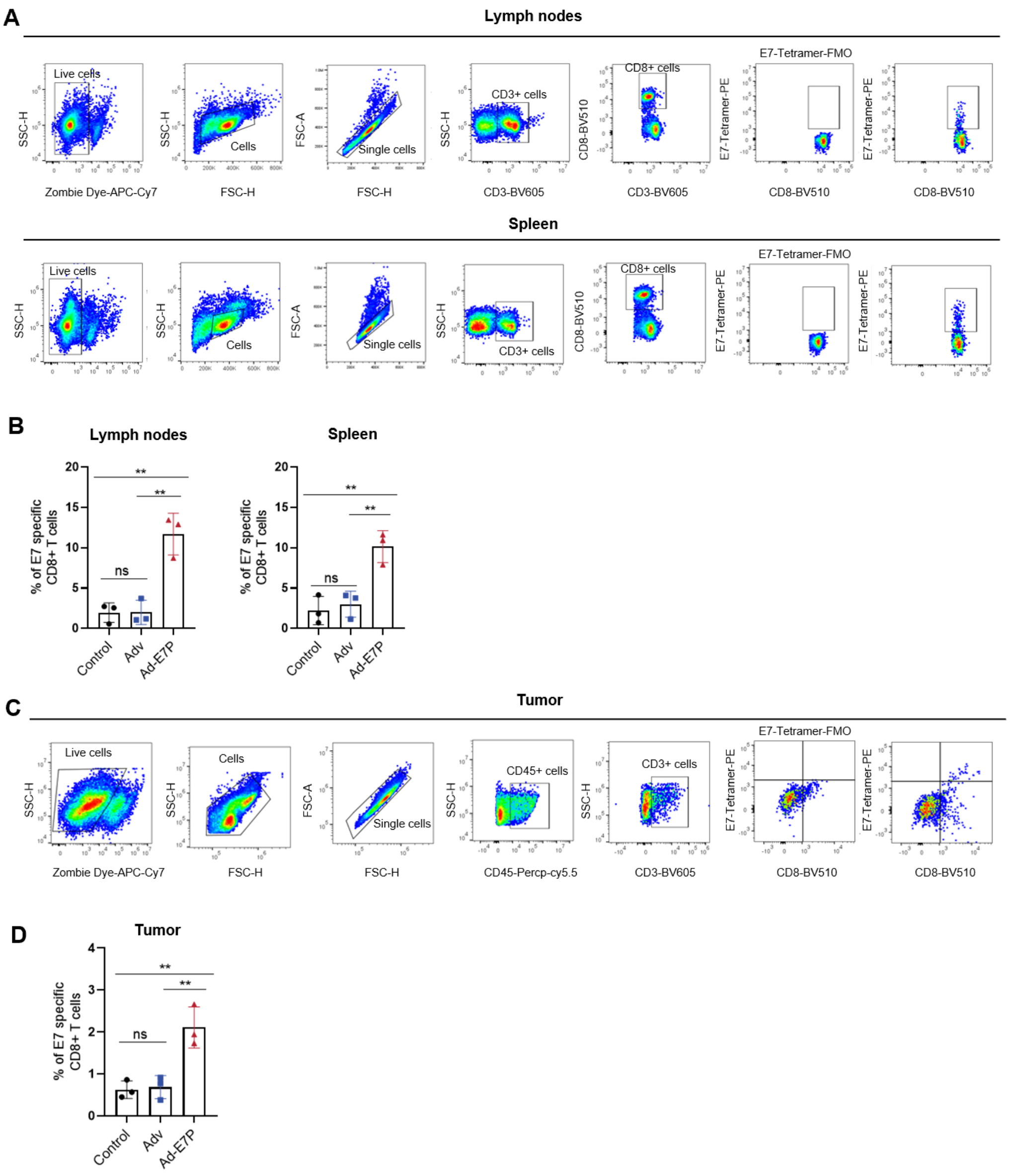

Figure S7. Identification of E7-specific T cell responses using tetramer staining.

(A and B) Gating strategy for identifying E7-specific CD8+ T cells in lymph nodes and spleens after vaccination (A), and quantification of E7-specific CD8+ T cells (B) (n = 3 per group). (C and D) Gating strategy for TC-1 tumors (C) and quantification of E7-specific CD8+ T cells (D). E7-tetramer FMO control was used to identify the positive population (n = 3 per group). Data are presented as the means ± SD. One-way analysis of variance (ANOVA) with Tukey's multiple comparisons test was performed for all comparisons (*$P < 0.05$, **$P < 0.01$, ***$P < 0.001$, ****$P < 0.0001$). *ns*, not significant.

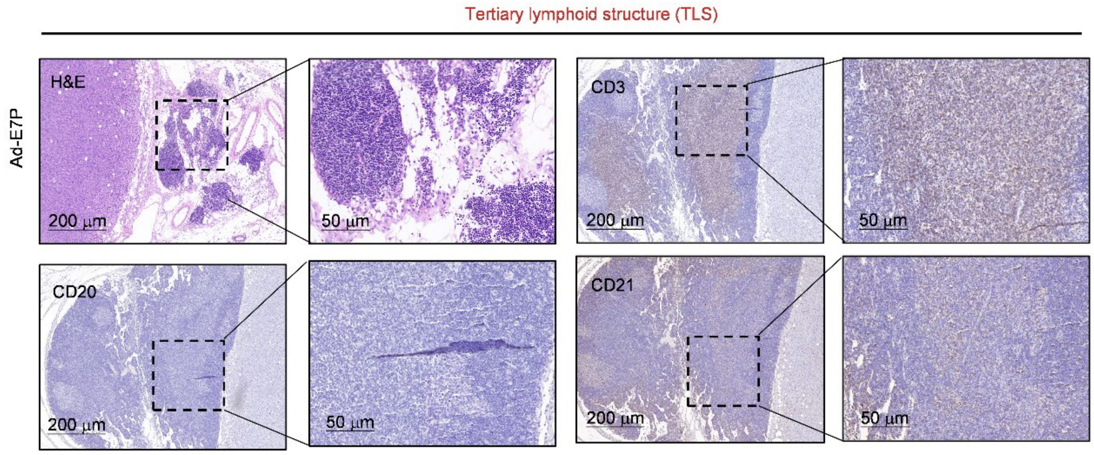

Figure S8. Tertiary lymphoid structures in tumor tissues.

Representative images of tertiary lymphoid structures (TLSs) detected in formalin-fixed paraffin-embedded TC-1 tumor sections by haematoxylin and eosin (H&E) staining (left) or by immunohistochemistry staining showing CD3+ T-cell zones, CD20+ B-cell zones, and CD21+ follicular dendritic cell (FDC) zones. Scale bars: 200 µm (overview) and 50 µm (zoomed-in view).

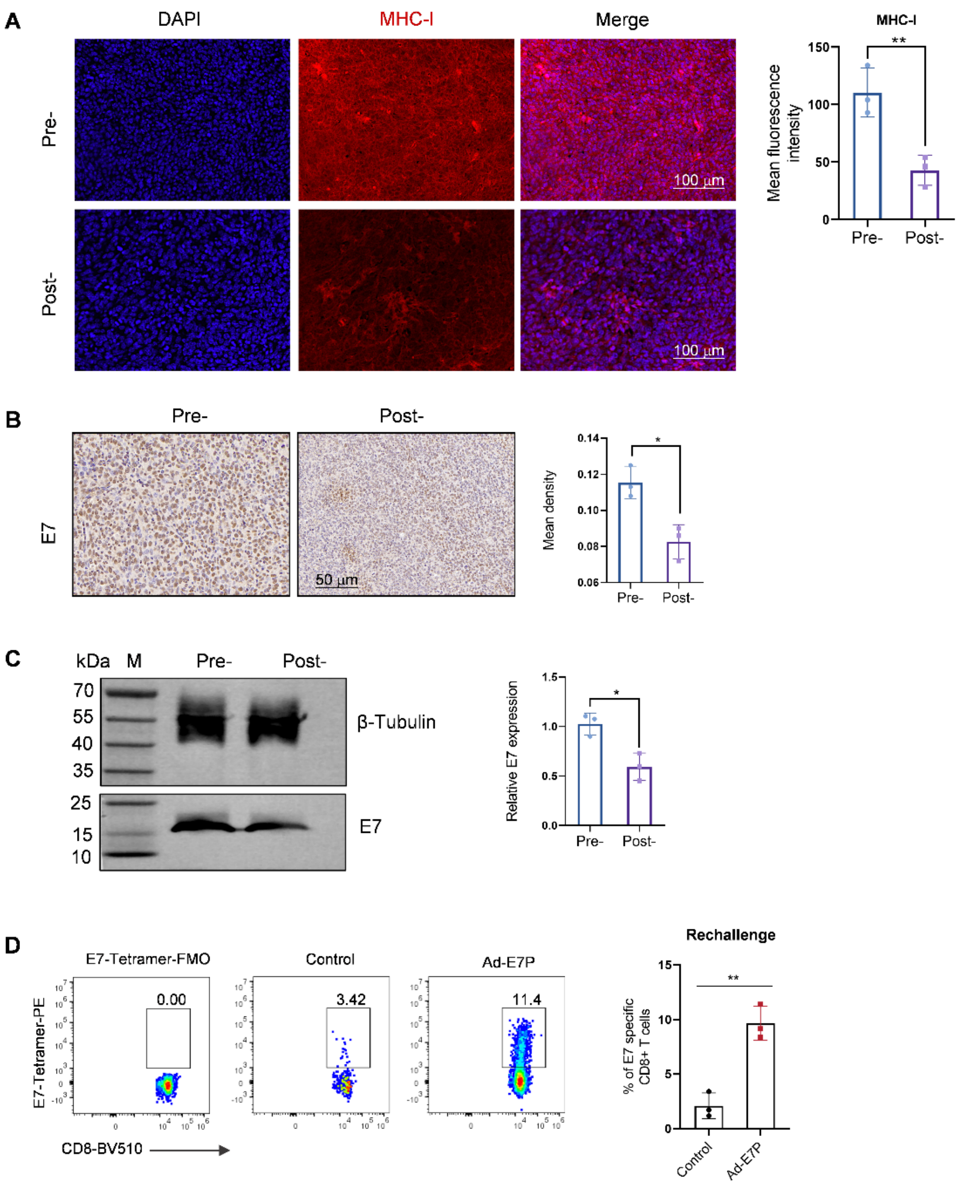

Figure S9. Decreased MHC-I and E7 expression contributes to TC-1 tumor relapse after vaccination.

(A and B) Representative images of immunofluorescence (A) and IHC (B) staining of paraffin-embedded sections of TC-1 tumor tissue at pre- (day 21 after the primary inoculation) and post-rechallenge (day 21 post-rechallenge) (*n* = 3 per group).

(C) Representative western blot image and quantitative analysis of E7 expression in TC-1 tumor tissue at pre- and post-rechallenge (*n* = 3 per group).

(D) Representative flow cytometry plots and quantification of E7-specific CD8+ T cells in spleens on day 7 after rechallenge ($n = 3$ per group).

Data are presented as the means ± SD. Two-tailed *t*-test was used for statistical analysis (*$P < 0.05$, **$P < 0.01$, ***$P < 0.001$, ****$P < 0.0001$). *ns*, not significant.

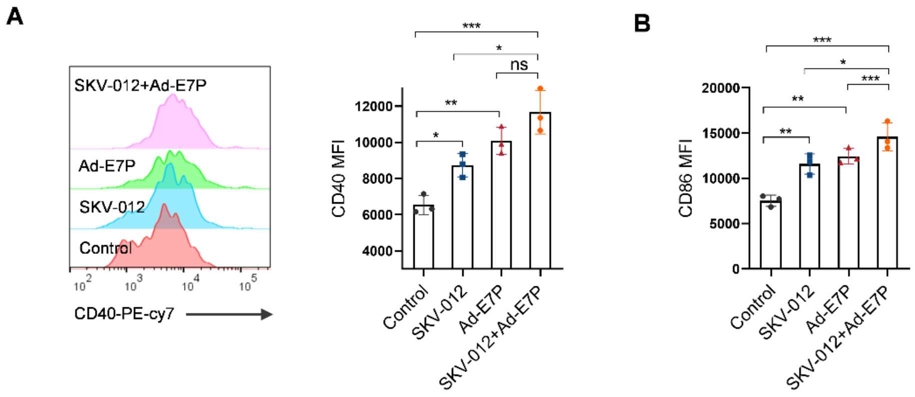

Figure S10. Characteristics of dendritic cells mature in lymph nodes and spleen.

(A) Representative flow cytometric analysis of CD40 expression on dendritic cells in lymph nodes (left), and quantification of mean fluorescence intensity (MFI) of CD40 expression (left) (n = 3 per group).

(B) Representative flow cytometric analysis of CD86 expression on dendritic cells in spleens (Right) (n = 3 per group).

Data are presented as the means ± SD. One-way analysis of variance (ANOVA) with Tukey's multiple comparisons test was performed for all comparisons (*P < 0.05, **P < 0.01, ***P < 0.001, ****P < 0.0001). *ns*, not significant.

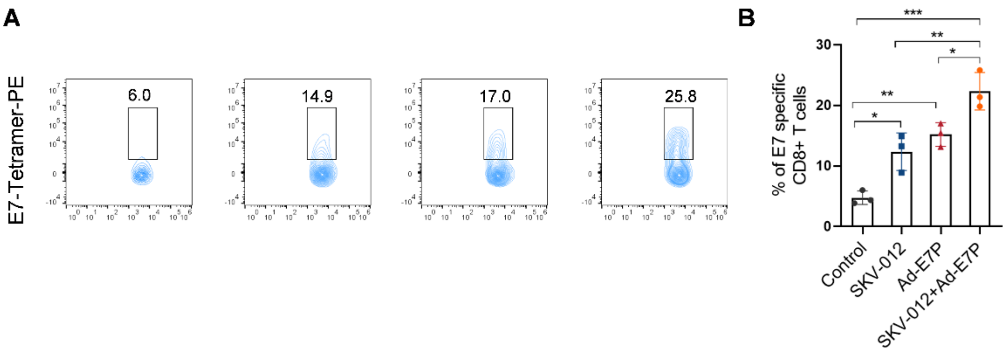

Figure S11. Tetramer staining of E7-specific cells in spleens.

(A) Representative flow cytometry plots are shown for E7-specific CD8+ T cells in spleens.

(B) Quantification of E7-specific CD8+ T cells using tetramer-based flow cytometry (*n* = 3 per group). (**P* < 0.05, ***P* < 0.01, ****P* < 0.001, *****P* < 0.0001). *ns*, not significant.

Data are presented as the means ± SD. One-way analysis of variance (ANOVA) with Tukey's multiple comparisons test was performed for all comparisons (**P* < 0.05, ***P* < 0.01, ****P* < 0.001, *****P* < 0.0001). *ns*, not significant.

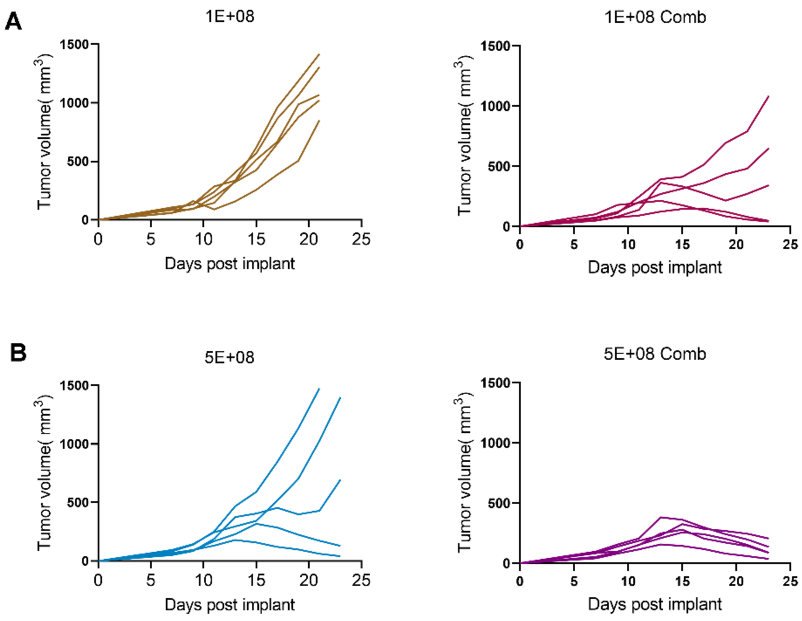

Figure S12. Vaccine dose-dependent synergy with oncolytic virus.

(A and B) Tumor growth curves (n = 5 per group). C57BL/6 mice were subcutaneously inoculated with 10^6 TC-1 tumor cells in the right flank. When tumor volumes reached approximately 50-100 mm^3, mice were received two reduced vaccine doses (1E+08 and 5E+08, respectively) of vaccine once a week and/or three doses of 10^6 PFU SKV-012 every two days. Mice were euthanized on days 23-25, as tumor volumes in the 1E+08 group approached the ethical endpoint of 1500 mm^3.

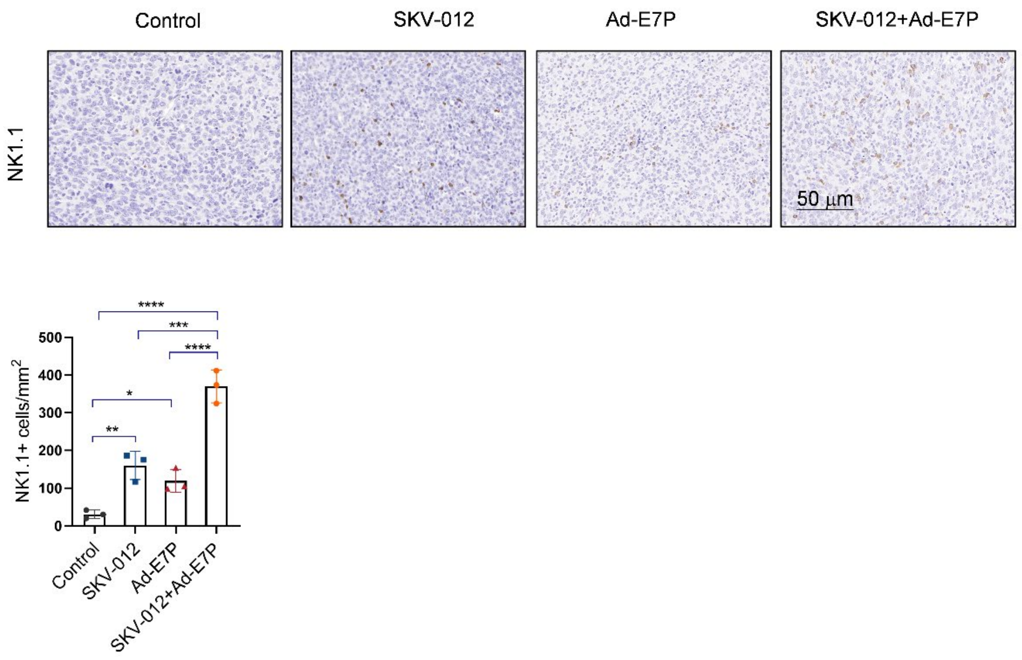

Figure S13. NK cell infiltration in TC-1 tumors.

Representative IHC staining images and corresponding quantification of NK1.1 in TC-1 tumor tissue after treatment (n = 3 per group). Scale bars, 50 μm. (*P < 0.05, **P < 0.01, ***P < 0.001, ****P < 0.0001). *ns*, not significant.

Data are presented as the means ± SD. One-way analysis of variance (ANOVA) with Tukey's multiple comparisons test was performed for all comparisons (*P < 0.05, **P < 0.01, ***P < 0.001, ****P < 0.0001). *ns*, not significant.

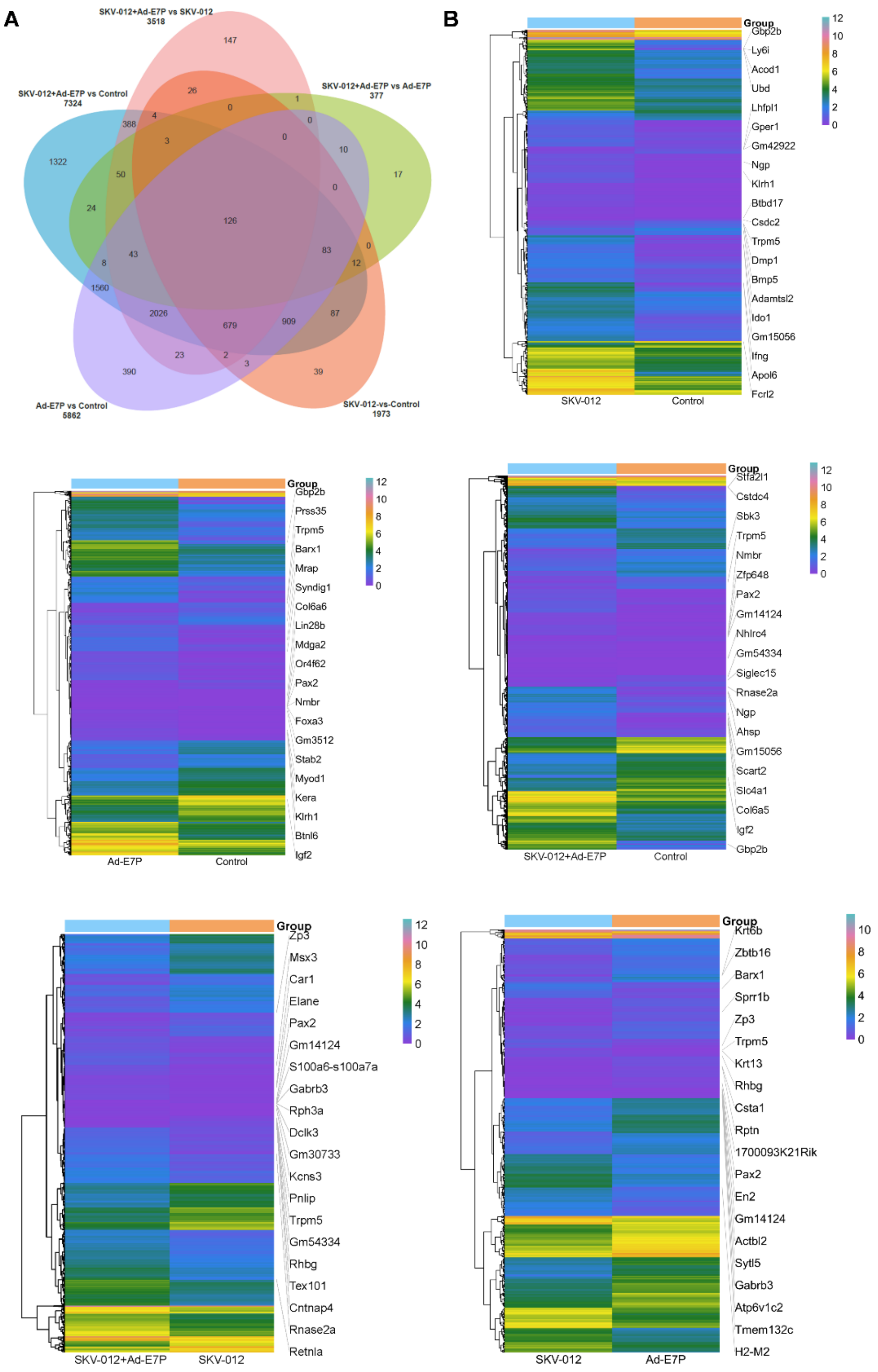

Figure S14. Differentially expressed genes in RNA-seq data.

(A and B) Venn diagram (A) and heatmap (B) of differentially expressed genes (DEGs) identified from RNA-seq analysis.

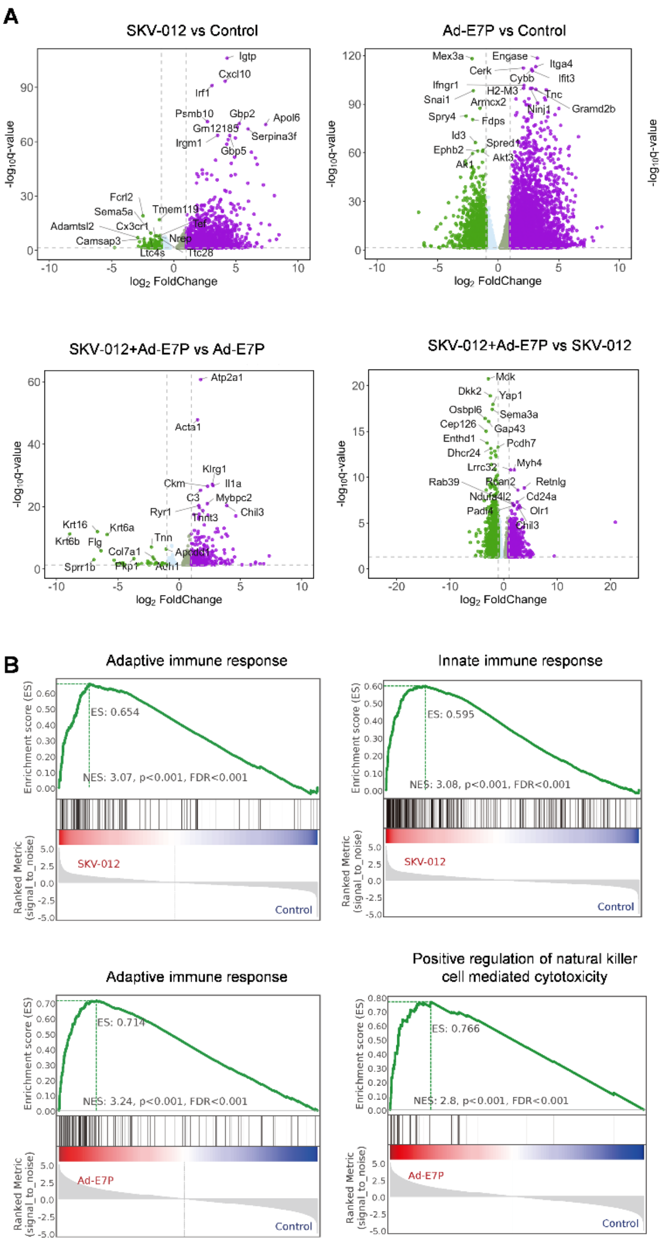

Figure S15. Volcano plot and GSEA analysis of transcriptomic changes.

(A) Volcano plot showing differentially expressed genes between the two groups. Green dots represent downregulated genes and purple dots represent upregulated genes.

(B) GSEA revealing enrichment of pathways related to the adaptive immune response, positive regulation of NK cell–mediated cytotoxicity, and innate immune activation after treatment.

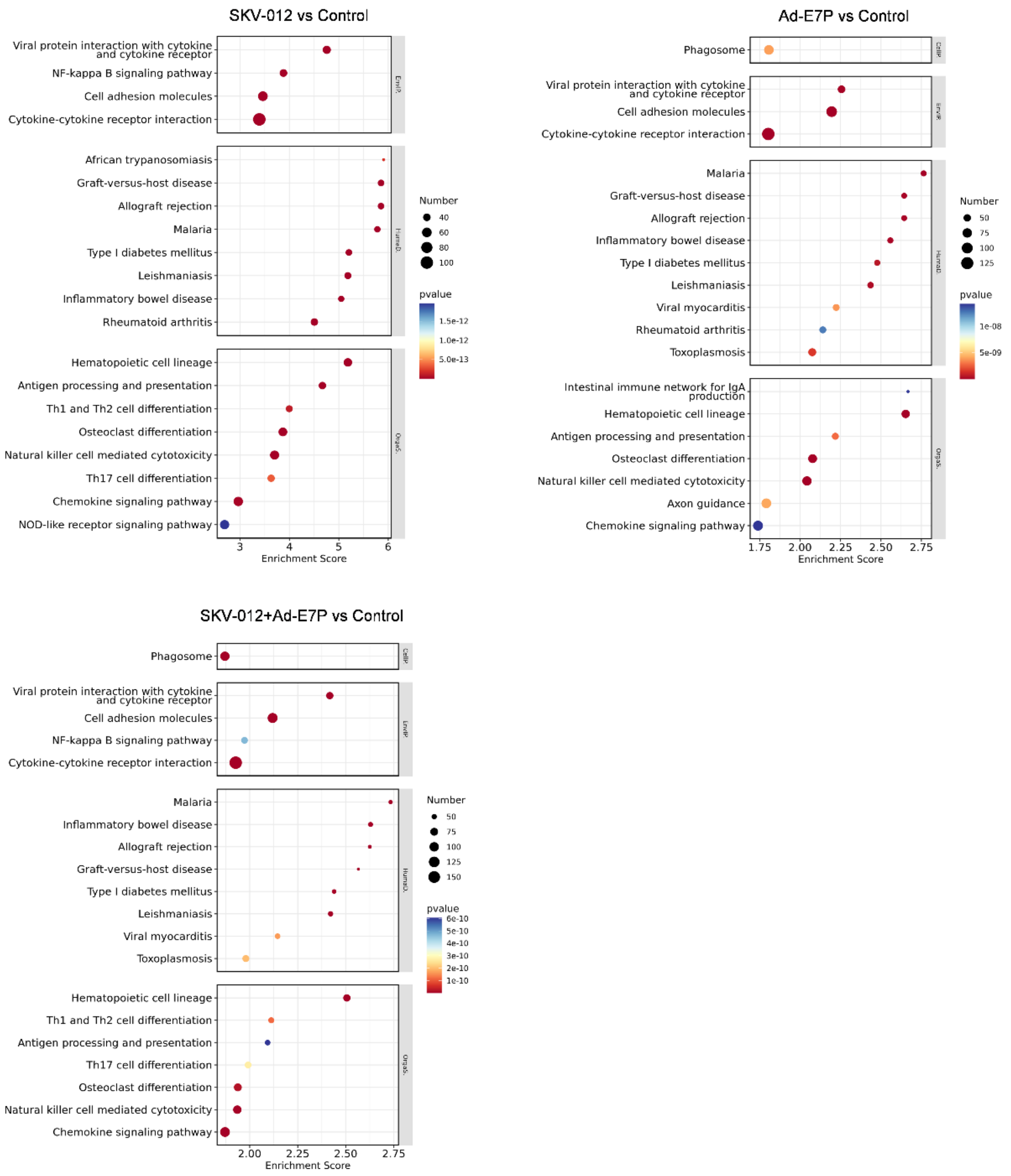

Figure S16. KEGG pathway enrichment bubble chart shows the top 20 enriched pathways (FDR < 0.05).

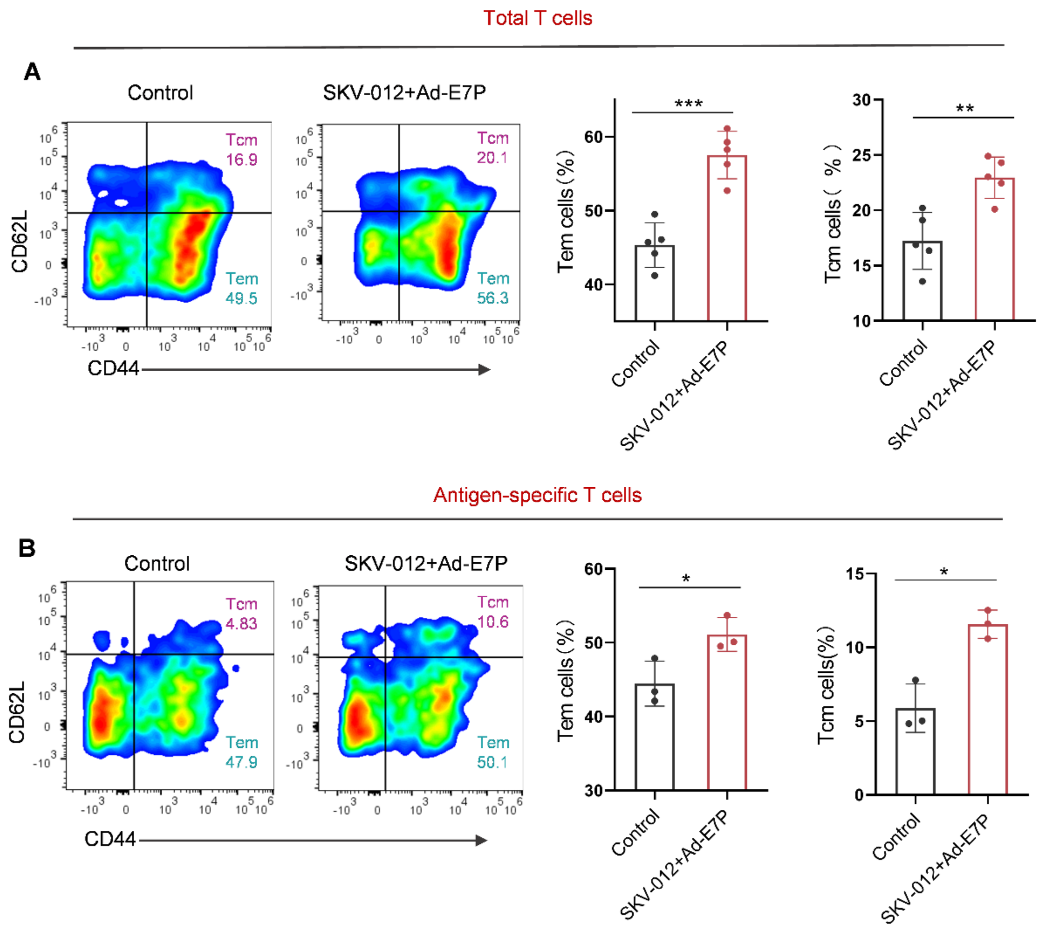

Figure S17. Evaluation of memory T-cell response.

(A and B) Representative dot plots showing total memory T cells (A, left) and antigen-specific memory CD8+ T cells (B, left) in splenic T cells at day 7 post–tumor challenge. Effector memory (Tem, (Tem, $CD44^{high}CD62L^{-}$) and central memory (Tcm, $CD44^{high}CD62L^{+}$) subsets are indicated, and the corresponding quantification of Tem and Tcm populations in the spleen is shown on the right (A, $n = 5$ per group; B, $n = 3$ per group).

Data are presented as the means ± SD. Two-tailed *t*-test was used for statistical analysis (*$P < 0.05$, **$P < 0.01$, ***$P < 0.001$, ****$P < 0.0001$). *ns*, not significant.

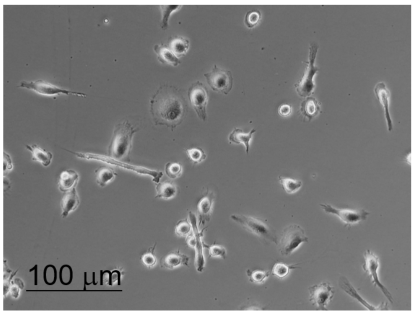

Figure S18. Dendritic cells (DCs) loaded with Ad-MP.

Microscopic image of Mo-DCs after Ad-MP loading. Scale bar, 100 μm.
